# Knockdown of a mucin‐like gene in *Meloidogyne incognita* (Nematoda) decreases attachment of endospores of *Pasteuria penetrans* to the infective juveniles and reduces nematode fecundity

**DOI:** 10.1111/mpp.12704

**Published:** 2018-10-22

**Authors:** Victor Phani, Tagginahalli N. Shivakumara, Keith G Davies, Uma Rao

**Affiliations:** ^1^ Division of Nematology ICAR‐Indian Agricultural Research Institute New Delhi 110012 India; ^2^ Department of Biological and Environmental Sciences University of Hertfordshire Hatfield AL10 9AB United Kingdom; ^3^ Norwegian Institute of Bioeconomy Research Ås 115, 1431 Norway

**Keywords:** attachment, fecundity, glycocalyx, immunity, *M. incognita*, mucin, *P. penetrans*

## Abstract

Mucins are highly glycosylated polypeptides involved in many host–parasite interactions, but their function in plant‐parasitic nematodes is still unknown. In this study, a mucin‐like gene was cloned from *Meloidogyne incognita* (*Mi‐muc‐1*, 1125 bp) and characterized. The protein was found to be rich in serine and threonine with numerous *O*‐glycosylation sites in the sequence. Quantitative real‐time polymerase chain reaction (qRT‐PCR) showed the highest expression in the adult female and *in situ* hybridization revealed the localization of *Mi‐muc‐1* mRNA expression in the tail area in the region of the phasmid. Knockdown of *Mi‐muc‐1* revealed a dual role: (1) immunologically, there was a significant decrease in attachment of *Pasteuria penetrans* endospores and a reduction in binding assays with human red blood cells (RBCs), suggesting that Mi‐MUC‐1 is a glycoprotein present on the surface coat of infective second‐stage juveniles (J2s) and is involved in cellular adhesion to the cuticle of infective J2s; pretreatment of J2s with different carbohydrates indicated that the RBCs bind to J2 cuticle receptors different from those involved in the interaction of *Pasteuria* endospores with Mi‐MUC‐1; (2) the long‐term effect of RNA interference (RNAi)‐mediated knockdown of *Mi‐muc‐1* led to a significant reduction in nematode fecundity, suggesting a possible function for this mucin as a mediator in the interaction between the nematode and the host plant.

## INTRODUCTION

The cuticle is a multi‐layered flexible structure in nematodes that acts as a hydrostatic exoskeleton and provides protection against the abiotic and biotic environment, including interactions with pathogenic microorganisms and, if parasitic, its hosts (Page, 2013). It is part of the extracellular matrix which maintains the nematode’s anatomical integrity and plays a crucial role in movement and growth (Lee, 2002). The cuticle, together with its surface coat, has been most extensively studied in *Caenorhabditis elegans* and, over the last several years, the nematode has become a model for the study of microbial pathogens (Ewbank, 2002; Sifri *et al*., 2005). Early research suggested that the cuticle and its surface coat were products of the hypodermis from which they were secreted (Wright, 1987); however, this view is now questionable as there is growing evidence that genes that play a role in the building of complex structures present in the cuticle’s surface coat are expressed in seam cells, and mutations to these genes are known to affect bacterial adhesion (Gravato‐Nobre *et al*., 2011).

The surface coat of animal‐parasitic nematodes contains glycans, which are thought to be influential in modulating the interaction with their hosts (Blaxter *et al*., 1992; Maizels *et al*., 2001; Politz and Philipp, 1992). Carbohydrate moieties on the surface of parasites are highly variable (Hicks *et al*., 2000; Theodoropoulos *et al*., 2001), and there are even variations among different populations of a particular species of parasite, having altered glycans as surface antigens or receptors (Appleton and Romaris, 2001; Haslam *et al*., 2000; Maass *et al*., 2007; Skelly and Wilson, 2006). The outermost cuticular layer appears to possess a number of specialized features and has been found to contain cuticulins, lipids, surface‐associated proteins, carbohydrates and probably glycoproteins (Blaxter *et al*., 1992; Page *et al*., 1992). Several biochemical and serological approaches used to characterize the surface coat properties of animal‐parasitic nematodes have revealed that rapid changes in the structure of the surface coat can occur between pre‐ and post‐parasitic forms (Proudfoot *et al*., 1993). Human red blood corpuscles have also been found to be useful in the study and characterization of the carbohydrate moieties present on the surface coat of plant‐parasitic nematodes (Spiegel and McClure, 1995; Spiegel *et al*., 1991).

Root‐knot nematodes (RKNs: *Meloidogyne* spp.) are economically one of the most damaging root pests and are responsible for extensive losses of staple food crops (Chitwood, 2003; Jones *et al*., 2013). These microscopic parasitic animals can manipulate plant defences and are thought to reprogram the cell cycle during the formation of specialized feeding cells (giant cells) in the roots for their continued nutrient supply (Gheysen and Mitchum, 2011). Owing to the lack of availability of effective management tools, and the global ban/restriction of many nematicides, alternative management strategies are being sought (Seid *et al*., 2015). Biological control offers the prospect to provide an alternative in an environmentally benign and self‐sustaining manner (Collange *et al*., 2011).

*Pasteuria penetrans* (Thorne) Sayre and Starr, a Gram‐positive, endospore‐forming soil bacterium of the *Bacillus*–*Clostridium* clade (Charles *et al*., 2005; Preston *et al*., 2003) has the potential to be developed into a commercial biological control agent for the management of RKN species [preferably the southern RKN *Meloidogyne incognita* (Kofoid and White) Chitwood] by turning the adult female cadaver into an ‘endospore sac’ (Davies, 2009; Stirling, 2014). When the cadaver eventually disintegrates, the bacterial endospores are released and lie dormant in the soil until they attach to the cuticle surface of the migrating pre‐parasitic second‐stage juveniles (J2s). They then germinate, by a process that is not understood, to form rhizoids which penetrate the developing female and eventually fragment to produce bacterial rods that proliferate and destroy the nematode’s reproductive capacity, ultimately resulting in the death of the adult female (Davies *et al*., 2011). The attachment of endospores to the cuticle of RKN J2s is likely to be governed by several factors and the cuticle surface coat plays a pivotal role in the attachment process (Davies and Danks, 1992; Spiegel and McClure, 1995).

Genome survey sequences of *P. penetrans* have been found to contain genes that encode collagen‐like proteins (Davies and Opperman, 2006), similar to those of *P. ramosa* (Moulton *et al*., 2009) and other endospore‐forming, animal‐parasitic *Bacillus* spp. (Sylvestre *et al*., 2003, 2005 ). It has been hypothesized that these collagen‐like proteins, which produce a hair‐like nap on the surface of the *Pasteuria* endospores, facilitate the attachment of the endospores to the nematode cuticle in a Velcro‐like fashion (Davies, 2009). Several investigations have shown that the nature of the attachment process is highly host specific, but is not linked to the phylogeny of the nematode (Davies *et al*., 2001). The nature of the cuticle receptor and the mechanism by which *Pasteuria* endospores bind are a matter of speculation; however, pretreatment of infective juveniles with a range of glycolytic enzymes and proteases suggests that surface coat glycans may help to explain the host specificity observed in the attachment of *Pasteuria* endospores to RKN cuticles (Davies and Danks, 1992; Davies and Redden, 1997; Davies *et al*., 1994).

Mucins are a family of high‐molecular‐weight proteins produced by the epithelial tissues in most organisms of the Kingdom Animalia (Marin *et al*., 2007). These proteins contain tandem repeats of amino acids rich in serine and threonine in their backbone which serve as sites for *O*‐glycosylation and have been suggested to play a role in immune defence (Hall and Altun, 2008). They are associated with both the innate and adapted immune systems and can be secreted or membrane bound to form a protective barrier over the epithelium (Loukas *et al*., 2000; Magalhães *et al*., 2010; Schulenburg *et al*., 2004; Strous and Dekker, 1992; Theodoropoulos *et al*., 2001). Membrane‐associated mucins are closely involved in the adhesion status of cells through electrostatic charge and steric effects of long chains protruding from the surface. The overabundance of certain membrane‐associated mucins suggests the role of the surface coat in the immune evasion of parasitic nematodes by changing the nematode surface cuticle adherence to defence cells (Gems and Maizels, 1996). Investigations using RNA interference (RNAi)‐mediated knockdown of mucin‐like proteins in *C. elegans* showed altered lectin binding to the surface coat (K. G. Davies *et al*., unpublished data), suggesting that it contains mucin‐like proteins amongst other glycosylated protein secretions (Gems and Maizels, 1996; Hemmer *et al*., 1991).

Although the roles of the majority of mucins in *C. elegans* are unknown, it is clear that *bah*, *bus* and *srf* mutants, which show altered bacterial adhesion, are glycosyltransferase mutants; glycosyltransferases are involved in the building of complex glycans on the surface coat of the cuticle (Gravato‐Nobre and Hodgkin, 2011). One of these mutations in *C. elegans*, *bus‐4*, which confers resistance to *Microbacterium nematophilum, Yersinia pestis* and *Y. pseudotuberculosis*, also shows altered mucin expression (Parsons *et al*., 2014). It has been hypothesized that mucin‐like peptides may also play a role in the attachment of *P. penetrans* endospores to the cuticle of RKNs (Davies, 2009; Davies and Curtis, 2011).

In this study, we identified and characterized a full‐length mucin‐like gene from the RKN *M. incognita*. In the absence of any tractable transformation tool for forward genetics screening, RNAi was used as a reverse genetics approach to assess the function of this gene (Dutta *et al*., 2015; Phani *et al*., 2017). Quantitative real‐time polymerase chain reaction (qRT‐PCR) was used to establish the transcriptional levels in different developmental stages of the nematode species and *in situ* hybridization was performed for the localization of mRNA expression in the body of the nematode. Knockdown of the mucin‐like gene by RNAi was employed and its effect on the attachment of *Pasteuria* endospores was studied; further characterization was investigated using red blood cells (RBCs) and inhibitory carbohydrates. Lastly, the role of the mucin‐like gene was also investigated for its effect on post‐infection nematode development and reproduction.

## RESULTS

### Molecular analysis, *in situ* hybridization and differential stage‐specific expression of *Mi‐muc‐1*

Based on the expressed sequence tag (EST) sequence (947 bp) of the mucin‐like gene from the cDNA library of *M. incognita*, the 1125‐bp full‐length cDNA sequence was amplified and cloned. The plasmid was named *Mi‐muc‐1*, encoding for a deduced 374‐bp amino acid sequence. The full‐length cDNA sequence was submitted to GenBank (Accession Number MG579969). Based on the analysis, the protein sequence was found to be rich in serine and threonine (26.2%) and contained numerous *O*‐glycosylation sites (Table S1, see Supporting Information).

**Figure 1:**
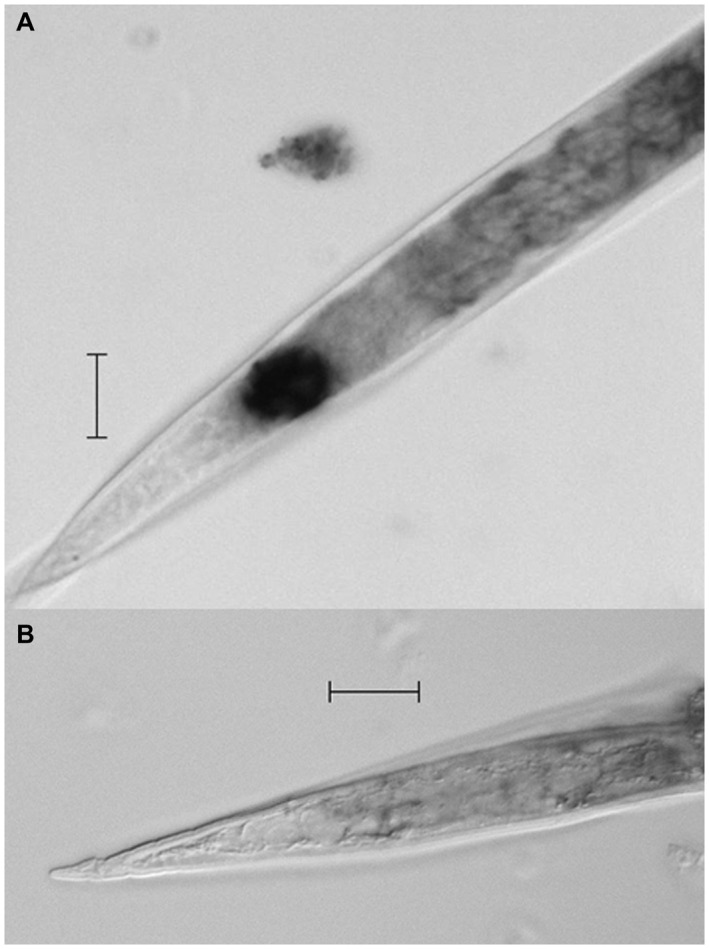
Localization of *Mi‐muc‐1* mRNA in *Meloidogyne incognita* second‐stage juveniles (J2s) by *in* s*itu* hybridization. (A) Hybridization of antisense digoxigenin (DIG)‐labelled cDNA probe shows the location in the phasmid area of the tail region. (B) Hybridization with DIG‐labelled sense cDNA probe was used as a control. Scale bar, 20 µm.

The localization of *Mi‐muc‐1* mRNA expression was determined by *in situ* hybridization in the pre‐parasitic J2s of *M. incognita*. The hybridization signal with digoxigenin (DIG)‐labelled antisense probe was detected in the tail region of the nematode, an area in which the phasmid is located (Fig. 1A); DIG‐labelled sense probes of the control group showed no such signal (Fig. 1B) and, similarly, no signal was detected in the anterior body part of the nematode with the DIG‐labelled antisense probe (Fig. S1, see Supporting Information). In addition, the localization of the mRNA expression of the other two homologues of *Mi‐muc‐1*, namely MucX and MucY, was also restricted to the tail region as for *Mi‐muc‐1* (Figs S2 and S3, see Supporting Information).

**Figure 2:**
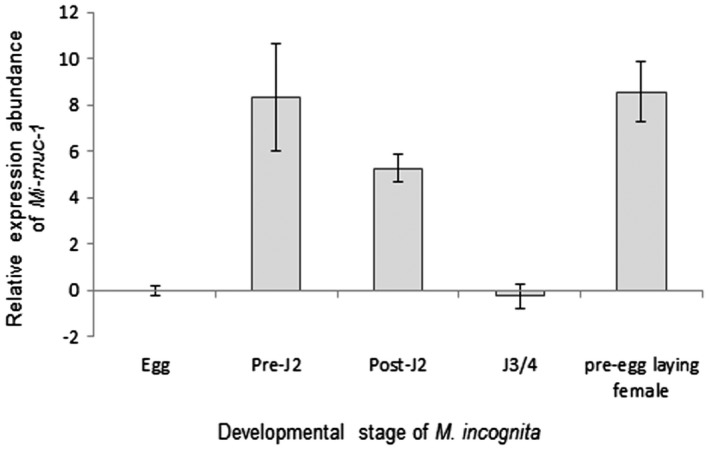
Relative expression abundance of *Mi‐muc‐1* in different developmental stages of *Meloidogyne incognita*. Using the 2^–ΔΔCT^ method, the relative expression level was quantified. Using the transcript level in eggs as a reference, the expression of *Mi‐muc‐1* was found to be highest in pre‐egg‐laying females, followed by pre‐parasitic second‐stage juveniles (J2s). Each bar represents the mean ± standard error (SE).

To determine the expression of *Mi‐muc‐1* in different life stages of *M. incognita*, qRT‐PCR was performed. Using the expression level in eggs as a reference, *Mi‐muc‐1* was found to be significantly (*P* < 0.05) upregulated in the pre‐parasitic J2s, parasitic J2s and pre‐egg‐laying females (Fig. 2). The highest level of expression was observed in the pre‐egg‐laying females, followed by the pre‐parasitic J2s, compared with insignificant expression in the third‐/fourth‐stage juveniles (J3s/J4s).

### Knockdown of *Mi‐muc‐1* and its effect on endospore and RBC attachment

The designed double‐stranded RNA (dsRNA) fragment resulted in the specific knockdown of *Mi‐muc‐1*. On BLAST search against the *M. incognita* database, two other mucin‐like sequences were retrieved which were amplified and cloned successfully (MucX and MucY). Alignment of the sequences revealed the percentage similarity of MucX and MucY to be 47.43% and 58.89%, respectively (at the nucleotide level), with *Mi‐muc‐1*. The result of qRT‐PCR showed no transcriptional alteration of MucX and MucY on knockdown of *Mi‐muc‐1* (Fig. S4, see Supporting Information).

**Figure 3:**
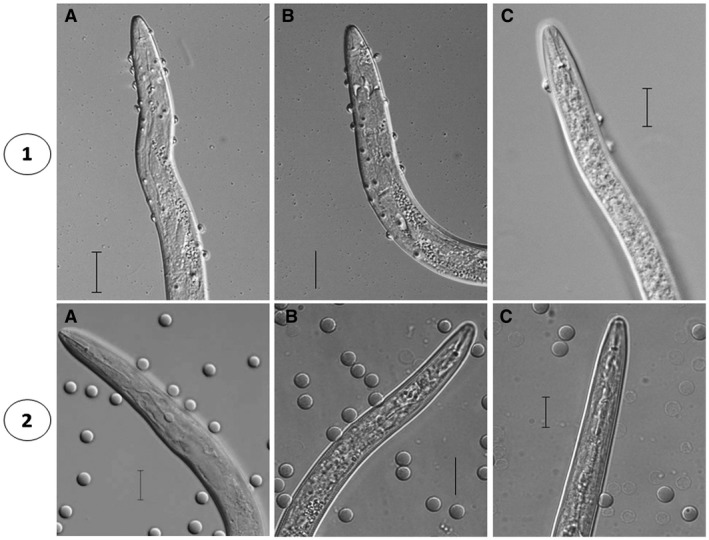
Effect of *Mi‐muc‐1* silencing on the attachment of endospores and red blood cells (RBCs) to *Meloidogyne incognita* second‐stage juveniles (J2s). (1) Decreased endospore attachment to cuticle surface on silencing of *Mi‐muc‐1*. (2) Decreased RBC adhesion to cuticle surface on silencing of *Mi‐muc‐1*. A, freshly hatched J2s; B, double‐stranded green fluorescent protein (dsGFP)‐treated J2s; C, *Mi‐muc‐1*‐silenced J2s. Scale bar, 20 µm.

The *in vitro* RNAi‐mediated knockdown of *Mi‐muc‐1* significantly (*P* < 0.05) reduced the adherence of endospores to the cuticle surface of *M. incognita* J2s. Approximately, 6 ± 2 endospores could attach to the target dsRNA‐treated worms, which was almost five times lower than that observed in the control groups. In comparison, the freshly hatched J2s (31 ± 6 endospores) and double‐stranded green fluorescent protein (dsGFP)‐treated J2s (30 ± 4 endospores) showed no significant difference in endospore adhesion (Fig. 3).

The knockdown of *Mi‐muc‐1* by dsRNA treatment was also found to cause significant (*P* < 0.05) reduction in adherence of RBCs to the cuticle surface. The average number of RBCs adhered to dsRNA‐treated juveniles was found to be 4 ± 3 vs. 12 ± 5 in freshly hatched J2s and 10 ± 6 in dsGFP‐treated J2s (Fig. 3), showing no significant difference in the last two cases.

### Assessment of endospore/RBC attachment on pre‐incubation in carbohydrate

**Figure 4:**
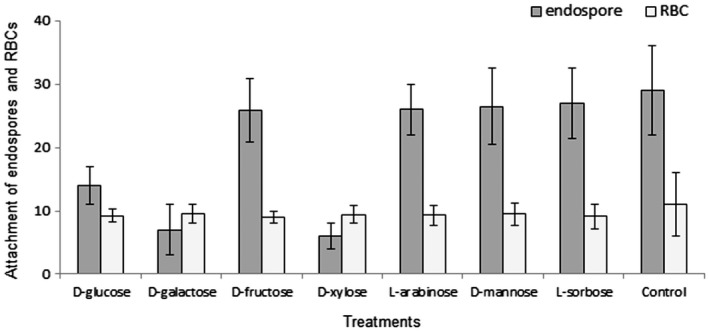
Attachment of endospores and red blood cells (RBCs) to second‐stage juvenile (J2) cuticle after incubation in different carbohydrates. d‐Glucose, d‐galactose and d‐xylose showed significantly (*P* < 0.05) less endospore attachment relative to d‐fructose, l‐arabinose, d‐mannose, l‐sorbose and control, whereas no statistically significant difference (*P* < 0.05) was observed for RBC attachment between different sugars.

The pre‐incubation of J2s in different carbohydrates affected the attachment of *P. penetrans* endospores to the juvenile cuticle surface of *M. incognita*. The average number of endospores attached to J2s is shown in Fig. 4. This resulted in a reduction of 51%, 75% and 79% in the presence of d‐glucose, d‐galactose and d‐xylose, respectively (Table 1), when compared with an average of 29 ± 7 endospores for the untreated control.

**Table 1:** Percentage decrease in endospore and RBC attachment over control after incubation in different carbohydrates (ANOVA endospore and RBC attachment; *P* < 0.05).

Sugars	Endospore attachment			RBC attachment		
		Treated	% change		Treated	% change
D‐glucose		14±3	51		9.2±1	8
D‐galactose		7±4	75		9.5±1.5	5
D‐fructose		25.9±5	11		9±1	10
D‐xylose		6±2	79		9.4±1.4	6
L‐arabinose		26±4	10		9.3±1.6	7
D‐mannose		26.5±6.1	9		9.5±1.8	5
L‐sorbose		27±5.5	7		9.1±1.9	9
Control		29±7	NA		11±5	NA

The binding of RBCs was also found to be affected by the incubation of J2s in carbohydrate solutions (Fig. 4); however, there was no statistically significant difference between any of the different sugars (Table 1).

Thus, it is evident that, although the attachment profile of endospores and RBCs is reduced by the use of different carbohydrate molecules, only in the case of *Pasteuria* endospores does this vary significantly (*P < *0.05) between the different sugars tested, with d‐glucose, d‐galactose and d‐xylose exhibiting the greatest effects.

### Evaluation of *M. incognita* development and *P. penetrans* establishment

**Figure 5:**
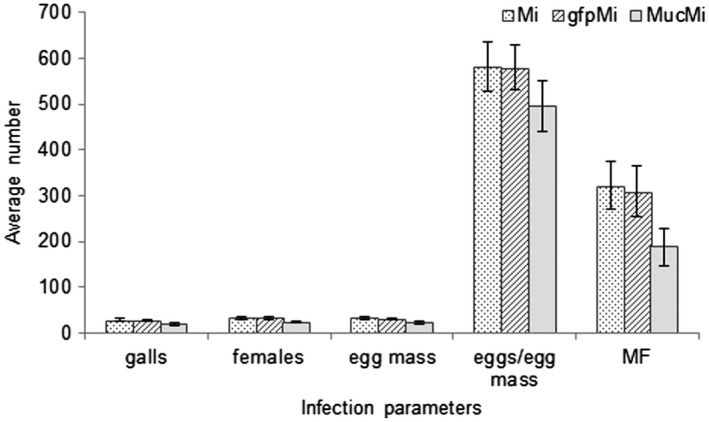
Assessment of parasitism on silencing of *Mi‐muc‐1*. The number of galls, adult females and egg mass per plant, eggs per egg mass and multiplication factor (MF) were reduced on silencing of *Mi‐muc‐1* in second‐stage juveniles (J2s) over controls. Mi, freshly hatched J2s; gfpMi, double‐stranded green fluorescent protein (dsGFP)‐treated J2s; MucMi, *Mi‐muc‐1*‐silenced J2s.

The long‐term effect of RNAi‐mediated knockdown of *Mi‐muc‐1* on the development and reproduction potential of *M. incognita* was assessed by infection bioassay using CYG germination pouches (Mega International, St Paul, MN, USA). At 30 days post‐inoculation (dpi), when the nematodes had completed their life cycle, dsRNA‐treated worms showed lower reproduction potential relative to control worms. A significant (*P* < 0.05) reduction was observed with respect to the number of galls, adult females, egg mass, eggs per egg mass and multiplication factor (MF) (Fig. 5). Approximately 29 ± 2.92, 28 ± 2.86 and 20 ± 2.25 galls developed per plant inoculated with freshly hatched, dsGFP‐treated and target dsRNA‐treated J2s, respectively. The average number of females developed per plant on infection with target dsRNA‐treated worms was 25 ± 2.84, whereas, for inoculation with freshly hatched and dsGFP‐treated worms, the numbers were 34 ± 3.93 and 33 ± 4.01, respectively. The average numbers of egg masses per plant produced by control worms (freshly hatched, 33 ± 3.46; dsGFP‐treated, 32 ± 2.73) were found to be significantly (*P* < 0.05) higher than that for the target dsRNA‐treated nematodes (23 ± 3.58). The approximate numbers of eggs per egg mass produced by freshly hatched, dsGFP‐treated and target dsRNA‐treated worms were found to be 582 ± 53, 579 ± 49 and 495 ± 56, respectively. MF (indicative of reproductive fitness and parasitic success) was reduced by approximately 40% in plants infected with *Mi‐muc‐1* dsRNA‐treated J2s, as compared with infection with freshly hatched and dsGFP‐treated J2s. Hence, silencing of *Mi‐muc‐1* in infective J2s retarded the development and reproduction potential of *M. incognita* in the host plant.

**Figure 6:**
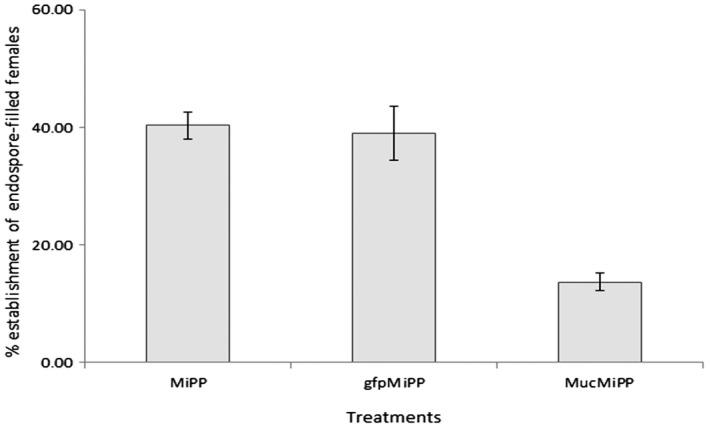
Percentage establishment of endospore‐filled females. The number of endospore‐filled females was reduced on *Mi‐muc‐1* silencing relative to controls. MiPP, endospore‐encumbered freshly hatched second‐stage juveniles (J2s); gfpMiPP, endospore‐encumbered double‐stranded green fluorescent protein (dsGFP)‐treated J2s; MucMiPP, endospore‐encumbered *Mi‐muc‐1*‐silenced J2s.

Further, the silencing of *Mi‐muc‐1* was found to exert a measurable effect on the development and establishment of *P. penetrans* inside adult *M. incognita* females. On infection with endospore‐encumbered target dsRNA‐treated J2s, the percentage of infected females was recorded as 13.66% ± 1.50%, which was significantly (*P* < 0.05) lower than that observed for the controls (Fig. 6). Inoculation with endospore‐encumbered freshly hatched (40.33% ± 2.33%) and dsGFP‐treated (39.01%±4.55%) J2s showed no significant difference.

## DISCUSSION

The results reported here demonstrate that the mucin‐like protein (Mi–MUC‐1) in *M. incognita* plays a dual role: (1) it facilitates the attachment of *P. penetrans* endospores to the cuticle of infective juveniles; and (2) it interferes with nematode development and fecundity. The present investigation is the first to identify and molecularly characterize a mucin‐like protein from a plant‐parasitic nematode with two functions. The attachment profiles of endospores and RBCs differed in the bioassays in which Mi‐MUC‐1 was involved and J2s were pretreated with a combination of different sugars. These results indicate the presence of more than one cellular attachment mechanism to the juvenile cuticle of *M. incognita*.

The reduction in endospore attachment on pre‐incubation in d‐glucose, d‐galactose and d‐xylose indicates that the ligands present on the nematode cuticle involved in attachment can recognize these sugars. Decreased attachment after incubation of the juveniles in carbohydrates, followed by washing, suggests that the sugar molecules chemically bind in a stable manner and block the receptors. The observation that the change in adhesion profile in response to different sugars and the interaction with knockdown of *Mi‐muc‐1* is different between endospores and RBCs indicates that Mi‐MUC‐1 is not the only cuticle receptor by which cells (endospore/RBC) can bind to the J2 cuticle, and suggests the availability of other biochemical ligands. This result updates the previous observations of Bird et al. (1989) and Davies and Danks (1992), in which the effect of the pre‐incubation of juveniles in different carbohydrates was tested for endospore attachment, and d‐xylose was found to reduce the attachment; however, in the later study, d‐glucose and d‐galactose did not show any effect.

The presence of carbohydrate residues on the surface of RKNs has been documented previously (Davies *et al*., 1988; Ibrahim, 1991; McClure and Zuckerman, 1982; Robertson *et al*., 1989; Spiegel and McClure, 1991) and carbohydrate recognition domains on the cuticle surface have also been predicted to interact with the *N*‐acetylglucosamine moieties on the spore surface (Davies and Danks, 1992). Interestingly, d‐glucose and d‐galactose were not found to exert any significant effect on endospore attachment in a previous study (Davies and Danks, 1992), whereas pre‐incubation in these two carbohydrates significantly reduced endospore attachment in the present investigation. This variation may be a result of differences in the biochemical composition of the endospore surface (Davies and Redden, 1997; Davies *et al*., 1994) or, indeed, the surface coat of J2s, which is also known to be dynamic and exhibits interspecific variation (Davies *et al*., 2001, 2008).

Compared with *Pasteuria* endospores, pretreatment of J2s with carbohydrates only had a marginal effect on the adhesion of RBCs, suggesting that different biochemical ligands are involved. This may be a result of the coevolutionary arms race between *P. penetrans* and its host nematode *M. incognita*, resulting in the high specificity observed in their attachment (Davies, 2009). This contrasts with the RBC binding assay, in which the interaction is likely to be much less specific, as RBCs and plant‐parasitic nematodes have not undergone prolonged coevolution. The reduced adherence of RBCs to the body surface on silencing of *Mi‐muc‐1* further strengthens the hypothesis of the occurrence of mucin‐like proteins on the cuticle. A dense coating of glycans stabilizes the RBCs (Evans and Graham, 1991; Eylar *et al*., 1962; Fernandes *et al*., 2011; Schauer, 2009) and aids in their attachment to other surfaces (Heidrich and Leutner, 1974; Winzler *et al*., 1967); the RBC glycoproteins may therefore aid in the stabilization of Mi‐MUC‐1 of *M. incognita* helping in their adhesion.

The results of *in situ* hybridization revealed that *Mi‐muc‐1* expression was localized in the tail region of *M. incognita* in an area similar to that identified by Premachandran et al. (1988) and Bellafiore et al. (2008) as the phasmid. Many non‐structural surface‐associated proteins on the nematode body surface are thought to be secreted from gland cells, namely amphids, pharyngeal glands, excretory pores, phasmids, hypodermis and seam cells (Gravato‐Nobre *et al*., 2011), and are subjected to continuous turnover (Blaxter and Robertson, 1998; Davies and Curtis, 2011). The constant sloughing off and renewal of these surface antigens protects the nematodes during movement in the soil environment as well as inside the host plant (Blaxter and Robertson, 1998; Curtis *et al*., 2011). The localization of *Mi‐muc‐1* expression around the phasmid is an indication that Mi‐MUC‐1 is secreted through the phasmidial pore and spreads over the cuticle surface. Interestingly, in *C. elegans*, in which seam cells are important in producing glycans to build complex surface carbohydrates (Gravato‐Nobre *et al*., 2011), a gene has been identified (*bus‐4*) that produces an altered mucin responsible for bacterial adhesion (Parsons *et al*., 2014). Some of these complex molecules present in the surface coat have also been documented in the subventral oesophageal glands in RKNs (Haegeman et al, 2013; Roze et al, 2008) and the internal excretory glands of the animal‐parasitic nematode *Toxocara canis* (Gems and Maizels, 1996; Hayes et al, 1990), and are therefore likely to have more than one role. Reduction in endospore attachment on silencing of *Mi‐muc‐1* suggests that the mucin‐like protein is involved in the facilitation of microbial adhesion on the surface of the nematode cuticle. Similar results have been observed previously (Derrien *et al*., 2010; Martin‐Sosa *et al*., 2002; Parsons *et al*., 2014; Ruiz‐Palacios *et al*., 2003; Sanchez *et al*., 2009), where mucins have been implicated to play a decisive role in the adhesion of microbial cells in *C. elegans* and humans.

*Mi‐muc‐1* was found to be expressed in pre‐parasitic (infective) J2s, parasitic J2s and females of *M. incognita*, indicating its presence in different phases of the nematode life cycle. The highest levels of expression were observed in pre‐parasitic and post‐parasitic J2s and pre‐egg‐laying females, with very little expression in eggs, J3 and J4 stages. The high expression of *Mi‐muc‐1* in pre‐egg‐laying females is indicative of its possible involvement in nematode reproduction and fecundity. Interestingly, this result is consistent with the observations of Ganji et al. (2014), where C‐type lectin (CTL) expression was found to be highest in sedentary females of *Rotylenchulus* *reniformis*, a semi‐endoparasitic nematode species. CTLs are one of the most prominent proteins, having a carbohydrate recognition domain which includes multiple calcium binding sites (Harcus *et al*., 2009), and have also been found to interfere with host immunity (Loukas and Maizels, 2000). The presence of CTLs on the surface of the marine nematode species, *Laxus oneistus*, is responsible for binding with the symbiotic bacteria necessary for nematode metabolism (Bulgheresi *et al*., 2006), and they have also been implicated in the bacterial infection of *C. elegans* (O’Rourke *et al*., 2006) and, more recently, in the recognition of bacterial, viral and fungal pathogens in other parasitic nematodes (Hoving *et al*., 2014). Therefore, the expression of *Mi‐muc‐1* in both the adult females and infective J2s suggests a dual function: (1) outside the plant host to help overcome the effects of pathogens and parasites taking part in the innate immune response (Hasnain *et al*., 2012; Loukas *et al*., 2000; Schulenburg *et al*., 2004; Theodoropoulos *et al*., 2001); and (2) inside the plant, where it may also play a role in the nematode–plant interaction, directly affecting its fecundity.

The reduced number of females and decreased egg production on *Mi‐muc‐1* dsRNA feeding suggests an additional role for the Mi‐MUC‐1 protein in affecting the fecundity of *M. incognita*. The reduction in the number of females on the root may be the result of either direct or indirect interactions: direct interactions may be because Mi‐MUC‐1 knockdown, which affects endospore and RBC adhesion, also has an effect on J2 mobility, thereby influencing root invasion; indirect effects may be via changes in the ability of the plant to recognize that it is undergoing parasitism by the nematode. The latter indirect effect provides evidence supporting the hypothesis of Kaplan and Davis (1987), who suggested that the nematode surface coat plays a role in plant–nematode interactions. The surface coat has been an active area of research for several decades and is thought to play a role in nematode–plant interactions, as it is known to be readily shed and replaced both outside and within the roots (Curtis *et al*., 2011; Davies and Curtis, 2011; Gravato‐Nobre *et al*., 1999; Lin and McClure, 1996; Spiegel and McClure, 1995). Interestingly, the most dominant model for the description of plant–pathogen interactions is the zig‐zag model of coevolution between microbial‐associated molecular patterns (MAMPs), secreted/excreted by the pathogen, and pattern recognition receptors (PRRs), which, in turn, ultimately leads to either a susceptibility or resistance response of the host (Jones and Dangl, 2006). The nematode surface coat could be a source of nematode effectors (Rosso and Grenier, 2011; Smant and Jones, 2011), which hitherto has provided a too narrowly defined bias (Cook *et al*., 2015) focusing on the identification and characterization of genes and proteins. A whole range of molecules have been ascribed the function of MAMPs; many of these include glycosides and have sugar‐based moieties, e.g. chitin, peptidoglycan and lipopolysaccharide (Iriti and Faoro, 2009). It has long been recognized that a component of the surface coat, nemin, is an elicitor of trapping devices in nematode predatory fungi. Recent research has characterized nemin as a member of a family of small lipophilic signalling molecules, ascarosides, which also appear to play a role in nematode–plant interactions (Manosalva *et al*., 2015). The ascarosides, as a group, consist of a glycoside with a lipophilic fatty acid side chain, and are only found in nematodes; it is interesting that fatty acid and retinol‐binding (FAR) proteins, which are also only found in nematodes and are associated with the nematode cuticle, appear to affect microbial adhesion to the cuticle and nematode interactions with the plant host (Phani *et al*., 2017).

Thus, in summary, we have demonstrated the presence of a mucin‐like protein in the cuticle surface coat of *M. incognita*, where it has two functions: (1) it plays a pivotal role in the attachment and interaction of *P. penetrans* endospores; and (2) it also appears to be indirectly involved in the nematode–plant interaction.

## EXPERIMENTAL PROCEDURES

### Nematode population

The single egg mass culture of an Indian isolate of *M. incognita* (Kofoid and White) Chitwood race 1 was multiplied on a susceptible tomato plant (*Solanum lycopersicum* L. cv. Pusa ruby) in a glasshouse at the ICAR‐Indian Agricultural Research Institute, New Delhi, India. Egg masses were manually picked from the infected roots and hatched via a modified Baermann’s assembly (Whitehead and Hemming, 1965). Freshly hatched J2s were used for experimental purposes.

### Bioinformatics

Fifteen genes encoding mucin‐like proteins in *C. elegans* were retrieved from the WormBase (version: WS260) database together with their translated products (Table S2, see Supporting Information). The protein sequences were analysed for their amino acid compositions and percentage serine and threonine content. The sequences were then subjected to BLAST search in WormBase Parasite (https://parasite.wormbase.org/Tools/Blast) and *Meloidogyne* genomic resources, INRA (https://www6.inra.fr/meloidogyne_incognita/Genomic-resources2/Blast) to retrieve the homologous sequences in *M. incognita*. The identities of the exact putative mucin‐like gene hits in *M. incognita* were obtained from the top scoring reciprocal BLAST hits (sequences with the smallest expected value and a large bit score). The return protein sequences were then stringently analysed for their percentage serine and threonine content using the ExPasy ProtParam tool (https://web.expasy.org/protparam/) and *O*‐glycosylation sites were predicted using the NetOGlyc 4.0 (https://www.cbs.dtu.dk/services/NetOGlyc/) server. Gene‐specific primers were designed (https://eu.idtdna.com/Primerquest/Home/Index) using customized parameters to amplify the partial sequence of the putatively assigned mucin‐like gene in *M. incognita*.

### Cloning, sequencing and characterization of mucin‐like gene in *M. incognita*

Total RNA was extracted from the freshly hatched J2s of *M. incognita* with a NucleoSpin^®^ RNA kit (Macherey‐Nagel, Düren, Germany) according to the manufacturer’s protocol, and cDNA was synthesized as described previously (Phani *et al*., 2017). A partial sequence of the putative mucin‐like gene (termed *Mi‐muc‐1*) was amplified from the cDNA using specific primers (Mi_muc_F and Mi_muc_R), cloned into the pGEM‐T Easy vector and sequenced via the Sanger sequencing method. To clone the full‐length *Mi‐muc‐1*, the partial sequence was subjected to BLAST search in WormBase Parasite to retrieve the corresponding coding sequence (CDS) transcript from the *M. incognita* genome (Abad *et al*., 2008). Accordingly, primers (InM2 and MR) were designed, PCR amplified, cloned and sequenced as described previously. Finally, the partial sequence and newly obtained sequence were aligned to obtain the complete *Mi‐muc‐1* sequence. Additional primers (MF and MR) were designed from ATG to TAA of the full‐length sequence, amplified from cDNA, cloned and sequenced for further confirmation. The primer details are given in Table 2.

**Table 2:** List of primers used in this study.

Primer names	Primer sequence (5′ ‐ 3′)	Product length (bp)	Tm (°C)
Mi_muc_F	GTAATCCTTACAGGCCCTTCTC	947	62
Mi_muc _R	TGACCCGTTGCACAATTACCGC		
InM2	CTACAACAACAACCTTACCAACT	428	62
MR	TTATTTTTTTGTCCCGAAATAACCGTTAGC		
MF	ATGTATAACGCCACATCTGGG	1125	62
MR	TTATTTTTTTGTCCCGAAATAACCGTTAGC		
M2_F	AGAGGATGGAAACACGTATGG	216	62
M2_R	CCTACCCACAGTGAACTGAAA		
Mucq_F	GCTCGGAGCTGAAGTTGTATTA	96	60
Mucq_R	GTGTTTGTTTGACACGCAGTTA		
dM2_F	GAGAGGATGGAAACACGTATGG	512	62
dM2_R	TGGCTGCGTTGTAGTTGTAG		
MucX_F	GGGAAGAGAAGAGCGTTATGG	799	62
MucX_R	AACGTGGACTCATGTGGAATAG		
MucY_F	CTACCGCTGAACCAACTACAA	655	62
MucY_R	GATTGACCCGTTGCACAATTAC		
qMucX_F	CTACGACAGAGGAACCAACTAAA	101	60
qMucX_R	ACAGAATGGAGGAGGCTTTG		
qMucY_F	TCTACCACGACGACCTTTAATTG	118	60
qMucY_R	GACATTCGCACACTCCTTGA		
gfp F	AGCGGCACGACTTCTTCA	750	60
gfp R	GTGTGGACAGGTAATGGTTGT		
18S_Mi_RT F	TCAACGTGCTTGTCCTACCCTGAA	115	60
18S_Mi_RT R	TGTGTACAAAGGGCAGGGACGTAA		
qMi‐actin F	TGACTCTGGAGATGGTGTTACG	142	60
qMi‐actin R	GTGATGACTTGACCGTCAGGC		
X_ISH_F	CCCGCTACTACAACAGAAGAAC	233	62
X_ISH_R	CAGAATGGAGGAGGCTTTGTAG		
Y_ISH_F	CTACCACCCTTCCAACAACAA	232	62
Y_ISH_R	GATTGACCCGTTGCACAATTAC		

Sequence homology was compared with the non‐redundant protein (nr) and nucleotide (nt) databases using BLASTX and BLASTN (https://blast.ncbi.nlm.nih.gov/Blast.cgi) allowing the low complexity regions in the algorithm parameter. The CDSs were predicted by the National Center for Biotechnology Information (NCBI) ORF Finder (https://www.ncbi.nlm.nih.gov/orffinder/) and conserved domains were analysed by the Conserved Domain Database in NCBI (https://www.ncbi.nlm.nih.gov/Structure/cdd/wrpsb.cgi). The ExPasy ProtParam tool (https://web.expasy.org/protparam/) and NetOGlyc 4.0 (https://www.cbs.dtu.dk/services/NetOGlyc/) server were used to analyse the protein sequence and the presence of *O*‐glycosylation sites.

### *In vitro* RNAi of mucin‐like gene in *M. incognita* J2s

A 512‐bp fragment, amplified from cDNA using specific primers (dM2_F and dM2_R), was used to synthesize the dsRNA of *Mi‐muc‐1* following the methodology described previously (Phani *et al*., 2017). Approximately 1500 freshly hatched *M. incognita* J2s were soaked in a solution with 0.1 mg/mL target dsRNA for *in vitro* RNAi, as described previously (Urwin *et al*., 2002). Soaking was continued for 15 h in the dark on a slowly moving rotator at 28 °C. Post‐incubation, the J2s were washed with molecular‐grade nuclease‐free water to remove any dsRNA contamination and total RNA was extracted from the dsRNA‐treated J2s using a NucleoSpin^®^ RNA kit (Macherey‐Nagel) following the manufacturer’s instructions. The RNA was reverse transcribed to cDNA and the level of transcript suppression after dsRNA feeding was quantified and analysed by qRT‐PCR. All soaking experiments were repeated thrice. dsRNA from a non‐endogenous gene (*gfp*, HF675000) was used as a non‐native negative control.

To confirm the specificity of dsRNA‐mediated knockdown of the target gene, the translated sequence of the full‐length *Mi‐muc‐1* was subjected to BLAST search against the *M. incognita* genome database using WormBase Parasite. The retrieved sequences with considerable homology (<1e‐10) were analysed for high serine and threonine content, and specific primers were designed for the amplification and cloning of the targets from cDNA. qRT‐PCR was performed to confirm the RNAi‐mediated silencing specificity of *Mi‐muc‐1*. dsGFP (HF675000) was used as a non‐native negative control. The primer details are provided in Table 2.

### Post‐RNAi phenotyping for *P. penetrans* endospore attachment

The endospores of *P. penetrans* (strain AII‐329, *Pasteuria* collection, ICAR‐IARI, New Delhi, India) were produced on adzuki bean [*Vigna angularis* (Willd.) Ohwi and Ohashi] with a single egg mass culture of *M. incognita*, as described previously (Rao *et al*., 2012). To check the effect of *Mi‐muc‐1* dsRNA treatment on endospore attachment to the J2 cuticle surface, approximately 200 dsRNA‐treated J2s were removed from the soaking solution, mixed with 100 μL of spore suspension (2.5 × 10^3^ mL^–1^) and centrifuged at 5000 *g* for 5 min (Hewlett and Dickson, 1993). The endospore adhesion was quantified by removing 30 J2s from the suspension; spore attachment was observed and photographed using a Zeiss, Jena, Germany Axiocam M2m compound microscope equipped with differential interference contrast (DIC) optics. Freshly hatched J2s and J2s soaked in dsGFP were used as controls. All assays were performed in triplicate.

### Post‐RNAi phenotyping for RBC attachment

To test the effect of *Mi‐muc‐1* dsRNA treatment on the attachment of human RBCs (kindly provided by Dr Sujaya Raghavendra for experimental purposes only) to the J2 cuticle surface, approximately 200 dsRNA‐treated J2s were removed from the soaking solution and placed in a 1.5‐mL microfuge tube. The solution was centrifuged at 5000 *g* for 5 min, the supernatant was removed and J2s were washed twice with Ringer’s solution. Following this, the J2s were topped with 50 μL of RBC suspension (in Ringer’s solution) (2.5 × 10^3^ mL^–1^) and centrifuged at 8000 *g* for 8 min. The adhesion of RBCs was quantified by removing around 30 J2s from the suspension; attachment was observed using a Zeiss Axiocam M2m compound microscope and photographed. Freshly hatched J2s and J2s soaked in dsGFP were used as controls. All assays were performed in triplicate.

### Pre‐incubation of *M. incognita* in carbohydrates and endospore/RBC attachment assay

To test the effect of incubation in different carbohydrates on the attachment of endospores and RBCs to the J2 cuticle surface, approximately 200 freshly hatched J2s were taken in a 1.5‐mL microfuge tube, centrifuged at 5000 *g* for 5 min and the supernatant was removed. The J2s were then resuspended in 200 µL of treatment solution and incubated at room temperature (28 °C) for 2 h on a slowly moving rotator. d‐Glucose, d‐galactose, d‐fructose, d‐xylose, l‐arabinose, d‐mannose and l‐sorbose were used for assay purposes, at 0.4 m concentration each, to prevent bursting of the nematode body. The treated J2s were then washed with double‐distilled water thrice and subjected for attachment study. Around 30 J2s were taken from each of the treatment solutions, and endospore and RBC attachments were measured. Photographs were taken using a Zeiss Axiocam M2m compound microscope and freshly hatched J2s were used as a control. All assays were performed in triplicate.

### *In situ* hybridization

Gene‐specific probes were used to localize the mRNA expression site of *Mi‐muc‐1* by *in situ* hybridization, as described in Kimber et al. (2002). Approximately 20 000 J2s were fixed in 2% paraformaldehyde at 4 °C for 18 h, followed by a 4‐h incubation at room temperature (28 °C). Specific primers (M2_F and M2_R; Table 2) were used to amplify the DIG‐labelled sense and antisense probes (Roche, Mannheim, Germany) from the cDNA of *M. incognita* pre‐parasitic J2s. DIG‐labelled sense or antisense probes were added separately to the hybridization solution containing the nematode sections, and then incubated at 50 °C for 12 h in a hybridization chamber. Following hybridization, the nematodes were stained and photomicrographs were taken with a Zeiss Axiocam M2m compound microscope. The experiment was repeated three times. In addition, *in situ* hybridization was also carried out for the *Mi‐muc‐1* homologues (MucX and MucY) to localize their mRNA expression site with specially designed primers (Table 2).

### Analysis of mRNA levels at different life stages of *M. incognita*

The expression patterns of *Mi‐muc‐1* in different developmental stages of *M. incognita* were analysed by qRT‐PCR with specially designed primers (Mucq_F and Mucq_R). For the extraction of the developmental stages of *M. incognita*, infection was performed on adzuki bean [*Vigna angularis* (Willd.) Ohwi and Ohashi] using CYG growth pouches (Mega International). The pouches were maintained in a growth chamber as described previously (Phani *et al*., 2017; Umarao *et al*., 2013) and various developmental stages were hand dissected at different intervals. Total RNA was extracted from approximately 1500 pre‐parasitic J2s, parasitic J2s (extracted between 4 and 6 dpi), J3s/J4s (extracted between 8 and 14 dpi) and pre‐egg‐laying females (extracted between 20 and 25 dpi), treated with RQ1 RNase‐free DNase (Promega, Madison, WI, USA) to remove genomic DNA contamination and finally reverse transcribed into cDNA. qRT‐PCR was performed in a realplex^2^ thermal cycler (Eppendorf, Hamburg, Germany) using a SYBR Green Supermix Kit (Eurogentec, Liege, Belgium), as described previously (Phani *et al*., 2017). 18S rRNA (HE667742) and actin (BE225475), two constitutively expressed genes, were used as references to normalize the gene expression level. Three biological and three technical replicates were maintained for each sample. The data were analysed by the ΔΔCt method (Livak and Schmittgen, 2001); the results were expressed as log_2_‐transformed fold change values and Student’s *t*‐test was performed. Primer details are provided in Table 2.

### Study of nematode development and *P. penetrans* establishment

To test the effect of *Mi‐muc‐1* knockdown on the reproduction potential of *M. incognita* and establishment of *P. penetrans*, infection was analysed in CYG growth pouches (Mega International) using adzuki bean as a susceptible host. The transfer of germinated seeds in pouches, maintenance for root proliferation, setting up of root infection and post‐infection maintenance of plants were conducted as described previously (Phani *et al*., 2017; Umarao *et al*., 2013). *Mi‐muc‐1* dsRNA‐treated J2s and endospore‐encumbered *Mi‐muc‐1* dsRNA‐treated J2s were used for inoculation purposes with five replications for each treatment. Freshly hatched J2s, endospore‐encumbered freshly hatched J2s, J2s treated with dsGFP and endospore‐encumbered dsGFP treated J2s were used as controls. The numbers of galls, adult females and egg masses produced per plant, number of eggs per egg mass and MF were considered as infection parameters to examine the reproduction potential of *M. incognita*. The percentage establishment of *P. penetrans* was calculated as [number of infected females/(number of infected females + number of uninfected females)] × 100, considering each pouch as a replicate.

### Statistical analyses

One‐way analysis of variance (ANOVA) and Tukey’s honestly significant difference (HSD) test (significance level at *P* < 0.05) were used to analyse the bioassay and expression data employing SAS version 9.3 for Windows (SAS Software, Inc. Cary, North Carolina, USA).

## Supporting information


**Fig. S1** The hybridization of digoxigenin (DIG)‐labelled sense (A) and antisense (B) cDNA probe of *Mi‐muc‐1* showed no signalling in the anterior body part of *Meloidogyne incognita* (scale bar, 20 µm).

**Fig. S2** The *in situ* hybridization result for MucX. The result showed no signalling in the anterior body part of *Meloidogyne incognita* with digoxigenin (DIG)‐labelled sense (A) and antisense (B) probes. The hybridization of antisense DIG‐labelled cDNA probe showed the location of mRNA expression in the phasmid area of the tail region (D) as compared with the control (C) (scale bar, 20 µm).

**Fig. S3** The *in situ* hybridization result for MucY. The result showed no signalling in the anterior body part of *Meloidogyne incognita* with digoxigenin (DIG)‐labelled sense (A) and antisense (B) probes. The hybridization of antisense DIG‐labelled cDNA probe showed the location of mRNA expression in the phasmid area of the tail region (D) as compared with the control (C). However, some non‐specific signals were also detected in the posterior body part (D) (scale bar, 20 µm).

**Fig. S4** (a) Sequence information of putative Mi‐muc‐1 homologues. (b) CLUSTAL 2.1 multiple sequence alignment. (c) Percent identity matrix (created by Clustal 2.1). (d) Quantitative real‐time polymerase chain reaction (qRT‐PCR) analysis shows no transcriptional alteration of MucX and MucY on downregulation of Mi‐muc‐1 (figure below) [double‐stranded green fluorescent protein (dsGFP) was used as a non‐native negative control].

**Table S1** The NetOGlyc prediction result. The output conforms to the GFF version 2 format (https://www.cbs.dtu.dk/services/NetOGlyc/). For the input sequence Mi‐MUC‐1, the server has provided a list of potential glycosylation sites, showing their positions in the sequence and the prediction confidence scores. Only the sites with scores higher than 0.5 are predicted as glycosylated and marked with the string ‘#POSITIVE’ in the comment field.

**Table S2** List of mucin‐like proteins of *Caenorhabditis elegans.*
