# Supplementary material for: Knockdown of a mucin‐like gene in Meloidogyne incognita (Nematoda) decreases attachment of endospores of Pasteuria penetrans to the infective juveniles and reduces nematode fecundity
Source: Mol Plant Pathol. 2018 Oct 22;19(11):2370–83. doi: 10.1111/mpp.12704 (PMC6638177; doi:10.1111/mpp.12704)

**Figure S1.** Hybridization of DIG-labeled sense (A) and antisense (B) cDNA probe of *Mi-muc-1* showed no signaling in the anterior body part of *M. incognita.* (scale bar: 20 µm)


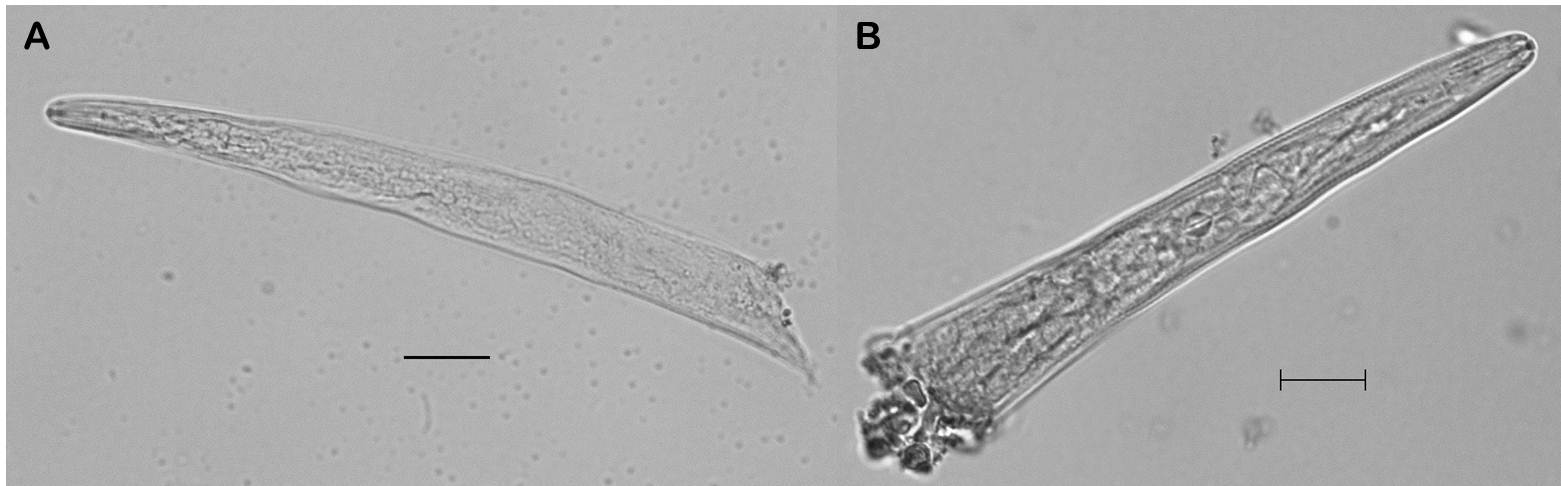

Supplement: Supplementary file 1 — Fig. S1 The hybridization of digoxigenin (DIG)‐labelled sense (A) and antisense (B) cDNA probe of Mi‐muc‐1 showed no signalling in the anterior body part of Meloidogyne incognita (scale bar, 20 µm). [file MPP-19-2370-s001.docx]
