# Supplementary material for: Knockdown of a mucin‐like gene in Meloidogyne incognita (Nematoda) decreases attachment of endospores of Pasteuria penetrans to the infective juveniles and reduces nematode fecundity
Source: Mol Plant Pathol. 2018 Oct 22;19(11):2370–83. doi: 10.1111/mpp.12704 (PMC6638177; doi:10.1111/mpp.12704)

**Figure S2.** The *in situ* hybridization result for MucX. The result showed no signaling in the anterior body part of *M. incognita* with DIG-labeled sense (A) and antisense (B) probes. The hybridization of antisense DIG-labeled cDNA probe showed the location of mRNA expression in the phasmidial area of tail region (D) as compared to control (C) (scale bar: 20 µm)


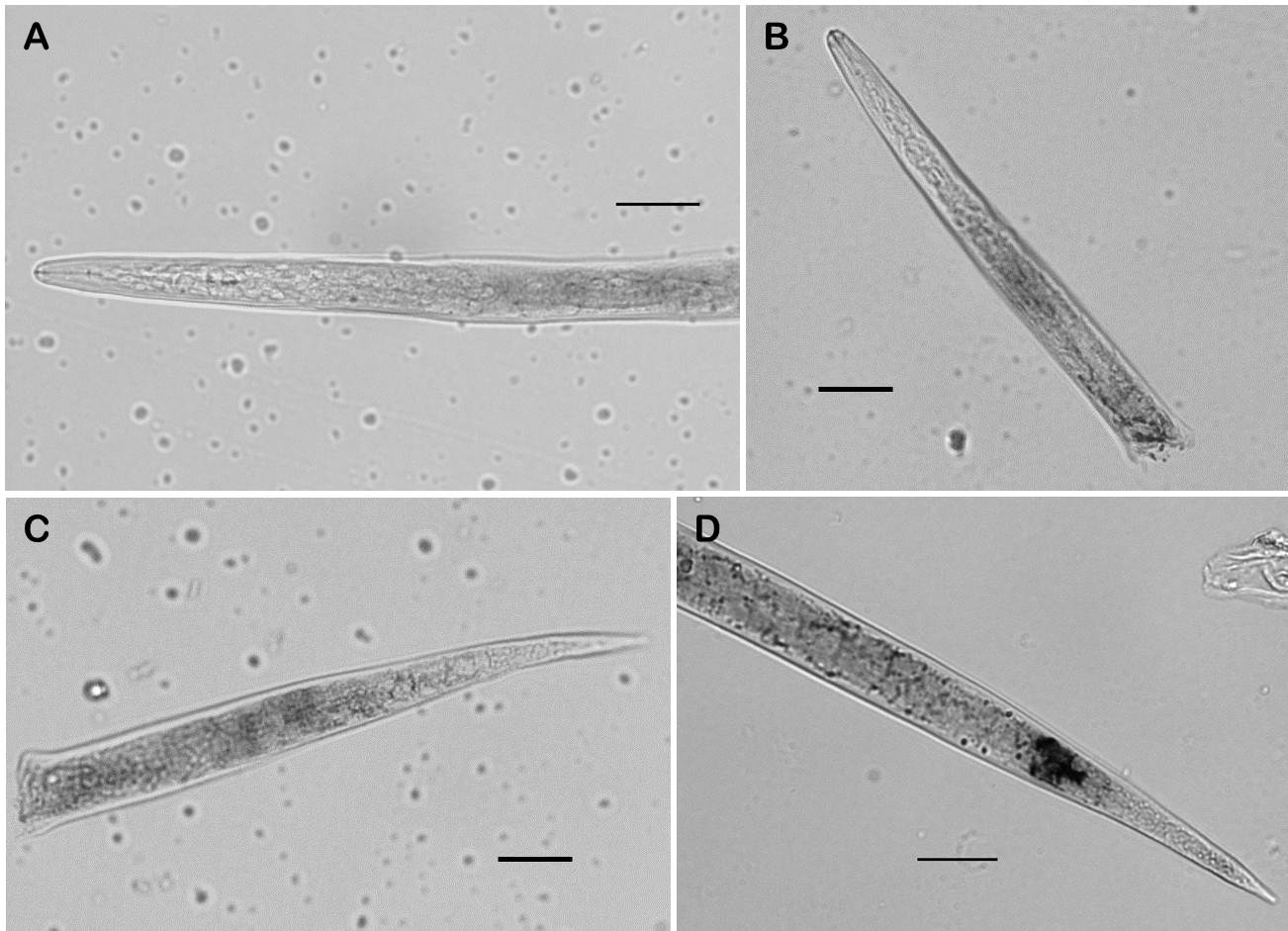

Supplement: Supplementary file 2 — Fig. S2 The in situ hybridization result for MucX. The result showed no signalling in the anterior body part of Meloidogyne incognita with digoxigenin (DIG)‐labelled sense (A) and antisense (B) probes. The hybridization of antisense DIG‐labelled cDNA probe showed the location of mRNA expression in the phasmid area of the tail region (D) as compared with the control (C) (scale bar, 20 µm). [file MPP-19-2370-s002.docx]
