# Supplementary material for: Knockdown of a mucin‐like gene in Meloidogyne incognita (Nematoda) decreases attachment of endospores of Pasteuria penetrans to the infective juveniles and reduces nematode fecundity
Source: Mol Plant Pathol. 2018 Oct 22;19(11):2370–83. doi: 10.1111/mpp.12704 (PMC6638177; doi:10.1111/mpp.12704)
Supplement: Supplementary file 4 — Fig. S4 (a) Sequence information of putative Mi‐muc‐1 homologues. (b) CLUSTAL 2.1 multiple sequence alignment. (c) Percent identity matrix (created by Clustal 2.1). (d) Quantitative real‐time polymerase chain reaction (qRT‐PCR) analysis shows no transcriptional alteration of MucX and MucY on downregulation of Mi‐muc‐1 (figure below) [double‐stranded green fluorescent protein (dsGFP) was used as a non‐native negative control]. [file MPP-19-2370-s004.docx]

**Figure S4**

1. **Sequence information of putative Mi-muc-1 homologues**

>MucX

GGGAAGAGAAGAGCGTTATGGAGGATTTCCCTTTAATGTTGGAGAACCGTTCGTTTTGGAAATTATTGCGGCACCAAAAAATATGATAATTGTTCATTTTAACTACAAACCATTCATAACATTTTCACGGGATGATTTGAGTAAATTGAGTTTATTGGGTGTTAGCTTGGCAATTGAACTTAGCTCTGTGGTTTTATGTCCTGATAAACCAATAACCACTACAATTGAACCAACTACAACAACAGAGGAACCAACTACTACAACAGAAGAACCAACTACTACAACAGAAGAACCAACTACTACAACAGAAGAACCCGCTACTACAACAGAAGAACCAACTACTACAACAGAAGAACCAACCACTACGACAGAGGAACCAACTACTACAACAGAAGAACCAACCACTACGACAGAGGAACCAACTACTACAACAGAAGAACCAACCACTACGACAGAGGAACCAACTAAAAACCCAACAACTACAACTAAGAAACCAACTACTACACTTCTACCAACCACAACCCCTACAAAGCCTCCTCCATTCTGTCCTGAACAAATTGTTCTAAATAACTTGGTTTAGAGTATATATAAAATAAGAAATATTTTTTGAAAATTTTAAAGAAAATTCCGGCAACTATCGATTTTGTGAAGCTCGGATTTGGAACTGGCTTTAGACGCCCAAAGCGCATTACAATCTTTGGAACACCTTTAGCACAACCTACAGCTTTTAACATACACATTGGTGAACCTGGTAAATTGTTAGTAAATGCTAATGTGCTATTCCACATGAGTCCACGTT

>MucY

CTACCGCTGAACCAACTACAACAACAACCGAGCCATCTACTACAACTACCGCTGAACCGACCACAACCACAACTGAACCCTCTACCACGACGACCTTTAATTGCGCTCAACCACCAGTCACATCATTGCTGAGTTACAACACTTCTGATCCCAAAATACAAACAGGTTCCAATGCAACTCAAGGAGTGTGCGAATGTCCAGCTGATCCAGGCAACAGTAATGTCTTTTTCATTCCTGTTACAACGATTACAACTGGTTCAAGTGCAAGTAATTCTTCAGTCATTATGAAATGTTCAAAAATGCAAGACTTCTGTATTTGCGACGAAGATGATATTTGTTGGAAAGTAATAAATGCTTATTCTCTAGTTGTGATTAATTCTTTCTGCGATCCAACAACCACAACAACCTTACCAACAACCACAACTACCACCCTTCCAACAACAACTACCACCCTCCCAACTACCACAACAACCTTGCCAACAACCACTACCACGTTGCCAACAACCACAACAACCTTACCAACTACCACTACCACCCTTCCAACTACCACAACTACCCTACCAACAACCACAACAAAGTTACAAACAACAACTACTACCCTACCTACAACAACACTTGCACCGTTAAATTGCGGTAATTGTGCAACGGGTCAATC

1. **CLUSTAL 2.1 multiple sequence alignment**

Sequence 1: MucX 799 bp

Sequence 2: MucY 655 bp

Sequence 3: Mi-muc-1 1125 bp

(Partial alignment)

Sequences (1:2) Aligned. Score: 30.3817

(Partial alignment)

Sequences (1:3) Aligned. Score: 20.776

(Partial alignment)

Sequences (2:3) Aligned. Score: 23.0534

MucX ------------------------------------------------------------

MucY ------------------------------------------------------------

Mi-muc-1 ATGTATAACGCCACATCTGGGGAGTGGAACGATTTATCATGCTTCCAAAAATTGGGTGGC

MucX -----------------GGGAAGAGAAGAGCGTTATGGAGGATTTCCCTTTAATGTTGGA

MucY -------------------CTACCGCTGAACCAACTACAACAACAACCGAGCCATCTACT

Mi-muc-1 GTCGTTTGTAAAAGAAATTGTACAGGAACATGTGGAAGTTCATCTATCAAGACTGCTTTA

:* .* :... : :. : *: :. * : ..: * :

MucX GAACCGTTCGTTTTGGAAATTATTGCGGCACCAAAAAATATGATAATTGTTCATTTTAAC

MucY ACAACTACCGCTGAACCGACCACAACCACAACTGAACCCTCTACCACGACGACCTTTAAT

Mi-muc-1 ATTACTAGCCCTTCCGACATTACGGACACAAGTTGTACATCGGGCAATTGGACTAAAAGA

. :.* : * * . * * .. .**. : .:.. : . .* .. :::*.

MucX -----------TACAAACCATTCATAACATTTTCACGGGATGATTTGAGTAAATTGAGTT

MucY -----------TGCGCTCAACCACCAGTCACATCATTG-CTGAGTTACAACACTTCTGAT

Mi-muc-1 GCAGGAGAGGATGGAAACACGTATGGGTATCAAATTGTAATGCAAGATTGGCTTAATTTT

*. ..:*.. . .. .: ::.: .**. : . . *: : :*

MucX TATTGGGTGTTAGCTTGG-CAATTGAACTTAGCTCTGTGGTTTTATGT---CCTGATAAA

MucY CCCAAAATACAAACAGGTTCCAATGCAACTCAAGGAGTGTGCGAATGT---CCAGCTGAT

Mi-muc-1 TATGAGGCAAATGCAAAATGTCTTGCGCTCGGAGCTGAAGTTGTATTAGTCCACAGCGTA

. ... . ::.*: . .:**... .. :*:. :** : *. . .::

MucX CCAATAACCACTACAATTGAACCAACTACAACAACAGAGGAACCAAC-------------

MucY CCAGGCAACAGTAATGTCTTTTTCATTCCTGTTACAACGATTACAAC-------------

Mi-muc-1 GCTGAAAACGAATTTATTCGTCAATTGTCAGCTCCATATATAACTGCGTGTCAAACAAAC

*:. .*.*. :: :.* : .: *:. :.** . .::.*:.*

MucX --------------------------------------------------TACTACAACA

MucY --------------------------------------------------TGGTTCAAGT

Mi-muc-1 ACATCGGTGTGTGCCACTATTTTCAGTTCACTGTGGGTAGGCTTGCACCGTAGTGCATTT

*. * **: :

MucX GAAGAACCAACTACTACAACAGAAGAACCAACTACTACAACAG-----------------

MucY G------CAAGTAATTCTTCAGTCATTATGAAATGTTCAAAAA-----------------

Mi-muc-1 TATCCTTCTTATAATTCTACTGTCGATTGTATCAATTCTGATGGAACTATCTGTGATTAT

*:: **.*:*::*:*:..:: * : *:*:..:.

MucX ----------------------------AAGAACCCGCTACTACAACAGAAGAACCAACT

MucY ----------------------------TGCAAGACTTCTGTATTTGCGACGAAGATGAT

Mi-muc-1 TTTAACATAACTGGAGGTCCTTCAGGGACAGAAACCGGAGGAGCATCTGGTCAACAAGAG

. ** .* :. :: *. ** .:..

MucX ACTACAACAGAAGAACCAACCACTACGACAGAGGAACCAACTACTACAACAG--------

MucY ATTTGTTGGAAAGTAATAAATGCTTATTCTCTAGTTGTGATTAATTCTTTCT--------

Mi-muc-1 GCTTGCGCTGCCATGTACAGCGCAACAACAGGACAGTGGAATGACATAGCATGCTTTAAC

. *: ....:. .* .*::. :*: . : .* *.. : : .

MucX -------------------------------------------------AAGAACCAACC

MucY -------------------------------------------------GCGATCCAACA

Mi-muc-1 AAATTAGGCGGTGTAATTTGTAAAAAGAACTGTTCGAACGCATGTGGAGTTGCATCAACA

*.: ****.

MucX ACTACGACAGAGGAACCAACTACTACAACAGA------AGAACCAACCACTACGACAGAG

MucY ACCACAACAACCTTACCAACAACCACAACT---------------ACCACCCTTCCAACA

Mi-muc-1 ACCACCACACAGTTGACTACAACTACAACGCAGCCAACTACAACAACAACCTTACCAACT

** ** *** . :..*:**:** ***** **.** .**..

MucX GAACCAACTAAAAACCCAACAACTACAACTAAGAAACCAACTACTACACTTCTACCAACC

MucY ACAACTACCACCCTCCCAACTACCACAACAACCTTGCCAACAACCACTACCACGTTGCCA

Mi-muc-1 ACCACTACCACCCTTCCAACTACCACAACTACCCTACCAACAACCACAACAAAGTTACAA

....*:** *...: *****:** *****:*. :.*****:** **:. . . ....

MucX ACAACCCCTACAAAGCCTCCTCCATTCTGTCCTGAACAAATTGTTCTAAATAACTTGGTT

MucY ACAACCACAACAACCTTACCAACTACCACTACCACCCTT---------------------

Mi-muc-1 ACAACAACTACTACCCTACCTACAACAACACTTGCACCGTTAAATTGCGGTAATTGTGCA

*****..*:**:*. :**:.*:: .: :. ...*

MucX TAGAGTATATATAAAATAAGAAATATTTTTTGAAAATTTTAAA-----------------

MucY ------------------------------------------------------------

Mi-muc-1 ACGGGTCAATCAAGAGTTATATATGATAAAAATACATTAACAAGTAATCGAATTGTTAGA

MucX ------------------GAAAATTCCGGCAACTATCGATTTTGTGAAGCTCGGATTTGG

MucY -------------------CCAACTACCACAACTACC-----------------------

Mi-muc-1 AATAAGGCTGCAAGTCAGTGCATCTTCAACTGCAAAGATCCAGATGTTTCACAGATTTG-

.*: * * .*:.*:*

MucX AACTGGCTTTAGACGCCCAAAGCGCA-TTACAATCTTTGGAACACCTTTAGCACAACCTA

MucY ------CTACCAACAACCACAACAAAGTTACAAAC-----AACAACTACTACCCTACCTA

Mi-muc-1 -CTACACTACTGATGCTGTAAGCGTCGCTAGAATTAACTGTACCGATCCTTCACAATTCT

**: .* .. :.*.*. . ** **: :**. .* : *.*:* :

MucX CAGCTTTT------------------------------AACATACACATTGGTGAACCTG

MucY CAAC----------------------------------AACACTTGCACCGTTAAATTGC

Mi-muc-1 GTGTTTGTGCTTCTAATGTTAAAAAAGGTTGCTATACAGTTACCAACACTGCACTTTTGG

:. .: * .** * : ::

MucX GTAAATTGTTAGTAAATGCTAATGTGCTATTCCACATGAGTCCACGTT

MucY GGTAATTG--------TGCAACGGGTCAATC-----------------

Mi-muc-1 CATATTATTCAATTTTTGCTAACGGTTATTTCGGGACAAAAAAATAA-

:*:*: ***:*. * ::*

**(c) Percent Identity Matrix (created by Clustal 2.1)**

1: MucX 100.00 46.36 47.43

2: MucY 46.36 100.00 58.89

3: Mi-muc-1 47.43 58.89 100.00

**(d) qRT PCR analysis shows no transcriptional alteration of MucX and MucY upon downregulation of Mi-muc-1 (Figure below)(dsGFP was used as non-native negative control)**
