# Supplementary material for: Knockdown of a mucin‐like gene in Meloidogyne incognita (Nematoda) decreases attachment of endospores of Pasteuria penetrans to the infective juveniles and reduces nematode fecundity
Source: Mol Plant Pathol. 2018 Oct 22;19(11):2370–83. doi: 10.1111/mpp.12704 (PMC6638177; doi:10.1111/mpp.12704)
Supplement: Supplementary file 5 — Table S1 The NetOGlyc prediction result. The output conforms to the GFF version 2 format (https://www.cbs.dtu.dk/services/NetOGlyc/). For the input sequence Mi‐MUC‐1, the server has provided a list of potential glycosylation sites, showing their positions in the sequence and the prediction confidence scores. Only the sites with scores higher than 0.5 are predicted as glycosylated and marked with the string ‘#POSITIVE’ in the comment field. [file MPP-19-2370-s005.docx]

**Table S1.** **The NetOGlyc prediction result.** The output conforms to the GFF version 2 format (http://www.cbs.dtu.dk/services/NetOGlyc/). For the input sequence Mi-MUC-1, the server has provided a list of potential glycosylation sites, showing their positions in the sequence and the prediction confidence scores. Only the sites with scores higher than 0.5 are predicted as glycosylated and marked with the string "#POSITIVE" in the comment field

**#seqname** **source feature start end score strand comment**

MI-MUC-1 netOGlyc-4.0.0.13 CARBOHYD 2 2 0.929722 . .#POSITIVE

MI-MUC-1 netOGlyc-4.0.0.13 CARBOHYD 4 4 0.765542 . .#POSITIVE

MI-MUC-1 netOGlyc-4.0.0.13 CARBOHYD 6 6 0.841229 . .#POSITIVE

MI-MUC-1 netOGlyc-4.0.0.13 CARBOHYD 16 16 0.717895 . .#POSITIVE

MI-MUC-1 netOGlyc-4.0.0.13 CARBOHYD 18 18 0.746828 . .#POSITIVE

MI-MUC-1 netOGlyc-4.0.0.13 CARBOHYD 25 25 0.932791 . .#POSITIVE

MI-MUC-1 netOGlyc-4.0.0.13 CARBOHYD 33 33 0.961639 . .#POSITIVE

MI-MUC-1 netOGlyc-4.0.0.13 CARBOHYD 34 34 0.978736 . .#POSITIVE

MI-MUC-1 netOGlyc-4.0.0.13 CARBOHYD 35 35 0.895084 . .#POSITIVE

MI-MUC-1 netOGlyc-4.0.0.13 CARBOHYD 37 37 0.964573 . .#POSITIVE

MI-MUC-1 netOGlyc-4.0.0.13 CARBOHYD 40 40 0.783144 . .#POSITIVE

MI-MUC-1 netOGlyc-4.0.0.13 CARBOHYD 43 43 0.90778 . . #POSITIVE

MI-MUC-1 netOGlyc-4.0.0.13 CARBOHYD 44 44 0.920892 . .#POSITIVE

MI-MUC-1 netOGlyc-4.0.0.13 CARBOHYD 52 52 0.969129 . .#POSITIVE

MI-MUC-1 netOGlyc-4.0.0.13 CARBOHYD 53 53 0.951929 . .#POSITIVE

MI-MUC-1 netOGlyc-4.0.0.13 CARBOHYD 57 57 0.918268 . .#POSITIVE

MI-MUC-1 netOGlyc-4.0.0.13 CARBOHYD 62 62 0.933642 . .#POSITIVE

MI-MUC-1 netOGlyc-4.0.0.13 CARBOHYD 65 65 0.930425 . .#POSITIVE

MI-MUC-1 netOGlyc-4.0.0.13 CARBOHYD 66 66 0.969007 . .#POSITIVE

MI-MUC-1 netOGlyc-4.0.0.13 CARBOHYD 67 67 0.928478 . .#POSITIVE

MI-MUC-1 netOGlyc-4.0.0.13 CARBOHYD 69 69 0.949767 . .#POSITIVE

MI-MUC-1 netOGlyc-4.0.0.13 CARBOHYD 78 78 0.968302 . .#POSITIVE

MI-MUC-1 netOGlyc-4.0.0.13 CARBOHYD 79 79 0.932915 . .#POSITIVE

MI-MUC-1 netOGlyc-4.0.0.13 CARBOHYD 81 81 0.889256 . .#POSITIVE

MI-MUC-1 netOGlyc-4.0.0.13 CARBOHYD 91 91 0.957061 . .#POSITIVE

MI-MUC-1 netOGlyc-4.0.0.13 CARBOHYD 93 93 0.914736 . .#POSITIVE

MI-MUC-1 netOGlyc-4.0.0.13 CARBOHYD 99 99 0.934625 . .#POSITIVE

MI-MUC-1 netOGlyc-4.0.0.13 CARBOHYD 100 100 0.968836 . .#POSITIVE

MI-MUC-1 netOGlyc-4.0.0.13 CARBOHYD 103 103 0.912209 . .#POSITIVE

MI-MUC-1 netOGlyc-4.0.0.13 CARBOHYD 105 105 0.830483 . .#POSITIVE

MI-MUC-1 netOGlyc-4.0.0.13 CARBOHYD 107 107 0.945135 . .#POSITIVE

MI-MUC-1 netOGlyc-4.0.0.13 CARBOHYD 114 114 0.919655 . .#POSITIVE

MI-MUC-1 netOGlyc-4.0.0.13 CARBOHYD 117 117 0.963448 . .#POSITIVE

MI-MUC-1 netOGlyc-4.0.0.13 CARBOHYD 118 118 0.978295 . .#POSITIVE

MI-MUC-1 netOGlyc-4.0.0.13 CARBOHYD 119 119 0.977176 . .#POSITIVE

MI-MUC-1 netOGlyc-4.0.0.13 CARBOHYD 122 122 0.98209 . . #POSITIVE

MI-MUC-1 netOGlyc-4.0.0.13 CARBOHYD 123 123 0.960326 . .#POSITIVE

MI-MUC-1 netOGlyc-4.0.0.13 CARBOHYD 126 126 0.915856 . .#POSITIVE

MI-MUC-1 netOGlyc-4.0.0.13 CARBOHYD 132 132 0.758568 . .#POSITIVE

MI-MUC-1 netOGlyc-4.0.0.13 CARBOHYD 133 133 0.766343 . .#POSITIVE

MI-MUC-1 netOGlyc-4.0.0.13 CARBOHYD 140 140 0.828945 . .#POSITIVE

MI-MUC-1 netOGlyc-4.0.0.13 CARBOHYD 141 141 0.833903 . .#POSITIVE

MI-MUC-1 netOGlyc-4.0.0.13 CARBOHYD 153 153 0.895251 . .#POSITIVE

MI-MUC-1 netOGlyc-4.0.0.13 CARBOHYD 154 154 0.733428 . .#POSITIVE

MI-MUC-1 netOGlyc-4.0.0.13 CARBOHYD 156 156 0.716006 . .#POSITIVE

MI-MUC-1 netOGlyc-4.0.0.13 CARBOHYD 160 160 0.949136 . .#POSITIVE

MI-MUC-1 netOGlyc-4.0.0.13 CARBOHYD 168 168 0.928214 . .#POSITIVE

MI-MUC-1 netOGlyc-4.0.0.13 CARBOHYD 169 169 0.905017 . .#POSITIVE

MI-MUC-1 netOGlyc-4.0.0.13 CARBOHYD 174 174 0.928949 . .#POSITIVE

MI-MUC-1 netOGlyc-4.0.0.13 CARBOHYD 192 192 0.940327 . .#POSITIVE

MI-MUC-1 netOGlyc-4.0.0.13 CARBOHYD 202 202 0.83886 . . #POSITIVE

MI-MUC-1 netOGlyc-4.0.0.13 CARBOHYD 204 204 0.901391 . .#POSITIVE

MI-MUC-1 netOGlyc-4.0.0.13 CARBOHYD 208 208 0.954151 . .#POSITIVE

MI-MUC-1 netOGlyc-4.0.0.13 CARBOHYD 210 210 0.969429 . .#POSITIVE

MI-MUC-1 netOGlyc-4.0.0.13 CARBOHYD 215 215 0.970502 . .#POSITIVE

MI-MUC-1 netOGlyc-4.0.0.13 CARBOHYD 216 216 0.908961 . .#POSITIVE

MI-MUC-1 netOGlyc-4.0.0.13 CARBOHYD 218 218 0.946934 . .#POSITIVE

MI-MUC-1 netOGlyc-4.0.0.13 CARBOHYD 221 221 0.959375 . .#POSITIVE

MI-MUC-1 netOGlyc-4.0.0.13 CARBOHYD 228 228 0.973721 . .#POSITIVE

MI-MUC-1 netOGlyc-4.0.0.13 CARBOHYD 229 229 0.976958 . .#POSITIVE

MI-MUC-1 netOGlyc-4.0.0.13 CARBOHYD 233 233 0.989561 . .#POSITIVE

MI-MUC-1 netOGlyc-4.0.0.13 CARBOHYD 234 234 0.992113 . .#POSITIVE

MI-MUC-1 netOGlyc-4.0.0.13 CARBOHYD 237 237 0.994793 . .#POSITIVE

MI-MUC-1 netOGlyc-4.0.0.13 CARBOHYD 238 238 0.988385 . .#POSITIVE

MI-MUC-1 netOGlyc-4.0.0.13 CARBOHYD 239 239 0.988448 . .#POSITIVE

MI-MUC-1 netOGlyc-4.0.0.13 CARBOHYD 240 240 0.990446 . .#POSITIVE

MI-MUC-1 netOGlyc-4.0.0.13 CARBOHYD 241 241 0.965712 . .#POSITIVE

MI-MUC-1 netOGlyc-4.0.0.13 CARBOHYD 243 243 0.983336 . .#POSITIVE

MI-MUC-1 netOGlyc-4.0.0.13 CARBOHYD 252 252 0.932954 . .#POSITIVE

MI-MUC-1 netOGlyc-4.0.0.13 CARBOHYD 259 259 0.907639 . .#POSITIVE

MI-MUC-1 netOGlyc-4.0.0.13 CARBOHYD 261 261 0.695174 . .#POSITIVE

MI-MUC-1 netOGlyc-4.0.0.13 CARBOHYD 263 263 0.744822 . .#POSITIVE

MI-MUC-1 netOGlyc-4.0.0.13 CARBOHYD 264 264 0.681762 . .#POSITIVE

MI-MUC-1 netOGlyc-4.0.0.13 CARBOHYD 269 269 0.839203 . .#POSITIVE

MI-MUC-1 netOGlyc-4.0.0.13 CARBOHYD 276 276 0.912288 . .#POSITIVE

MI-MUC-1 netOGlyc-4.0.0.13 CARBOHYD 281 281 0.957031 . .#POSITIVE

MI-MUC-1 netOGlyc-4.0.0.13 CARBOHYD 282 282 0.980434 . .#POSITIVE

MI-MUC-1 netOGlyc-4.0.0.13 CARBOHYD 284 284 0.93506 . . #POSITIVE

MI-MUC-1 netOGlyc-4.0.0.13 CARBOHYD 286 286 0.974379 . .#POSITIVE

MI-MUC-1 netOGlyc-4.0.0.13 CARBOHYD 287 287 0.945464 . .#POSITIVE

MI-MUC-1 netOGlyc-4.0.0.13 CARBOHYD 290 290 0.828091 . .#POSITIVE

MI-MUC-1 netOGlyc-4.0.0.13 CARBOHYD 299 299 0.5 . . #POSITIVE

MI-MUC-1 netOGlyc-4.0.0.13 CARBOHYD 303 303 0.840366 . .#POSITIVE

MI-MUC-1 netOGlyc-4.0.0.13 CARBOHYD 313 313 0.979932 . .#POSITIVE

MI-MUC-1 netOGlyc-4.0.0.13 CARBOHYD 314 314 0.972681 . .#POSITIVE

MI-MUC-1 netOGlyc-4.0.0.13 CARBOHYD 315 315 0.911431 . .#POSITIVE

MI-MUC-1 netOGlyc-4.0.0.13 CARBOHYD 317 317 0.966143 . .#POSITIVE

MI-MUC-1 netOGlyc-4.0.0.13 CARBOHYD 318 318 0.983352 . .#POSITIVE

MI-MUC-1 netOGlyc-4.0.0.13 CARBOHYD 321 321 0.98468 . . #POSITIVE

MI-MUC-1 netOGlyc-4.0.0.13 CARBOHYD 325 325 0.981237 . .#POSITIVE

MI-MUC-1 netOGlyc-4.0.0.13 CARBOHYD 326 326 0.938179 . .#POSITIVE

MI-MUC-1 netOGlyc-4.0.0.13 CARBOHYD 328 328 0.835886 . .#POSITIVE

MI-MUC-1 netOGlyc-4.0.0.13 CARBOHYD 333 333 0.892462 . .#POSITIVE

MI-MUC-1 netOGlyc-4.0.0.13 CARBOHYD 337 337 0.938978 . .#POSITIVE

MI-MUC-1 netOGlyc-4.0.0.13 CARBOHYD 339 339 0.870105 . .#POSITIVE

MI-MUC-1 netOGlyc-4.0.0.13 CARBOHYD 341 341 0.937319 . .#POSITIVE

MI-MUC-1 netOGlyc-4.0.0.13 CARBOHYD 345 345 0.898179 . .#POSITIVE

MI-MUC-1 netOGlyc-4.0.0.13 CARBOHYD 349 349 0.766032 . .#POSITIVE

MI-MUC-1 netOGlyc-4.0.0.13 CARBOHYD 351 351 0.82614 . . #POSITIVE

MI-MUC-1 netOGlyc-4.0.0.13 CARBOHYD 364 364 0.901975 . .#POSITIVE

MI-MUC-1 netOGlyc-4.0.0.13 CARBOHYD 368 368 0.814256 . .#POSITIVE

MI-MUC-1 netOGlyc-4.0.0.13 CARBOHYD 370 370 0.599663 . .#POSITIVE

MI-MUC-1 netOGlyc-4.0.0.13 CARBOHYD 372 372 0.722562 . .#POSITIVE

MI-MUC-1 netOGlyc-4.0.0.13 CARBOHYD 378 378 0.945612 . .#POSITIVE

MI-MUC-1 netOGlyc-4.0.0.13 CARBOHYD 380 380 0.905143 . .#POSITIVE

MI-MUC-1 netOGlyc-4.0.0.13 CARBOHYD 381 381 0.937314 . .#POSITIVE

MI-MUC-1 netOGlyc-4.0.0.13 CARBOHYD 382 382 0.875854 . .#POSITIVE

MI-MUC-1 netOGlyc-4.0.0.13 CARBOHYD 383 383 0.920448 . .#POSITIVE

MI-MUC-1 netOGlyc-4.0.0.13 CARBOHYD 387 387 0.898866 . .#POSITIVE

MI-MUC-1 netOGlyc-4.0.0.13 CARBOHYD 388 388 0.973156 . .#POSITIVE

MI-MUC-1 netOGlyc-4.0.0.13 CARBOHYD 392 392 0.906558 . .#POSITIVE

MI-MUC-1 netOGlyc-4.0.0.13 CARBOHYD 394 394 0.922426 . .#POSITIVE

MI-MUC-1 netOGlyc-4.0.0.13 CARBOHYD 398 398 0.870356 . .#POSITIVE

MI-MUC-1 netOGlyc-4.0.0.13 CARBOHYD 403 403 0.756863 . .#POSITIVE

MI-MUC-1 netOGlyc-4.0.0.13 CARBOHYD 404 404 0.598901 . .#POSITIVE

MI-MUC-1 netOGlyc-4.0.0.13 CARBOHYD 411 411 0.888308 . .#POSITIVE

MI-MUC-1 netOGlyc-4.0.0.13 CARBOHYD 414 414 0.884212 . .#POSITIVE

MI-MUC-1 netOGlyc-4.0.0.13 CARBOHYD 418 418 0.957561 . .#POSITIVE

MI-MUC-1 netOGlyc-4.0.0.13 CARBOHYD 419 419 0.977211 . .#POSITIVE

MI-MUC-1 netOGlyc-4.0.0.13 CARBOHYD 420 420 0.980974 . .#POSITIVE

MI-MUC-1 netOGlyc-4.0.0.13 CARBOHYD 421 421 0.960521 . .#POSITIVE

MI-MUC-1 netOGlyc-4.0.0.13 CARBOHYD 423 423 0.938231 . .#POSITIVE

MI-MUC-1 netOGlyc-4.0.0.13 CARBOHYD 426 426 0.972406 . .#POSITIVE

MI-MUC-1 netOGlyc-4.0.0.13 CARBOHYD 427 427 0.983608 . .#POSITIVE

MI-MUC-1 netOGlyc-4.0.0.13 CARBOHYD 429 429 0.991047 . .#POSITIVE

MI-MUC-1 netOGlyc-4.0.0.13 CARBOHYD 430 430 0.969121 . .#POSITIVE

MI-MUC-1 netOGlyc-4.0.0.13 CARBOHYD 432 432 0.975969 . .#POSITIVE

MI-MUC-1 netOGlyc-4.0.0.13 CARBOHYD 435 435 0.966502 . .#POSITIVE

MI-MUC-1 netOGlyc-4.0.0.13 CARBOHYD 436 436 0.978084 . .#POSITIVE

MI-MUC-1 netOGlyc-4.0.0.13 CARBOHYD 438 438 0.925033 . .#POSITIVE

MI-MUC-1 netOGlyc-4.0.0.13 CARBOHYD 441 441 0.93735 . . #POSITIVE

MI-MUC-1 netOGlyc-4.0.0.13 CARBOHYD 443 443 0.944257 . .#POSITIVE

MI-MUC-1 netOGlyc-4.0.0.13 CARBOHYD 447 447 0.978346 . .#POSITIVE

MI-MUC-1 netOGlyc-4.0.0.13 CARBOHYD 448 448 0.976261 . .#POSITIVE

MI-MUC-1 netOGlyc-4.0.0.13 CARBOHYD 450 450 0.933 . . #POSITIVE

MI-MUC-1 netOGlyc-4.0.0.13 CARBOHYD 452 452 0.965716 . .#POSITIVE

MI-MUC-1 netOGlyc-4.0.0.13 CARBOHYD 456 456 0.974764 . .#POSITIVE

MI-MUC-1 netOGlyc-4.0.0.13 CARBOHYD 457 457 0.934704 . .#POSITIVE

MI-MUC-1 netOGlyc-4.0.0.13 CARBOHYD 459 459 0.954769 . .#POSITIVE

MI-MUC-1 netOGlyc-4.0.0.13 CARBOHYD 462 462 0.949799 . .#POSITIVE

MI-MUC-1 netOGlyc-4.0.0.13 CARBOHYD 468 468 0.930067 . .#POSITIVE

MI-MUC-1 netOGlyc-4.0.0.13 CARBOHYD 470 470 0.953829 . .#POSITIVE

MI-MUC-1 netOGlyc-4.0.0.13 CARBOHYD 472 472 0.930456 . .#POSITIVE

MI-MUC-1 netOGlyc-4.0.0.13 CARBOHYD 474 474 0.98972 . . #POSITIVE

MI-MUC-1 netOGlyc-4.0.0.13 CARBOHYD 477 477 0.990629 . .#POSITIVE

MI-MUC-1 netOGlyc-4.0.0.13 CARBOHYD 478 478 0.993809 . .#POSITIVE

MI-MUC-1 netOGlyc-4.0.0.13 CARBOHYD 480 480 0.986686 . .#POSITIVE

MI-MUC-1 netOGlyc-4.0.0.13 CARBOHYD 481 481 0.986388 . .#POSITIVE

MI-MUC-1 netOGlyc-4.0.0.13 CARBOHYD 482 482 0.986359 . .#POSITIVE

MI-MUC-1 netOGlyc-4.0.0.13 CARBOHYD 483 483 0.985276 . .#POSITIVE

MI-MUC-1 netOGlyc-4.0.0.13 CARBOHYD 488 488 0.964272 . .#POSITIVE

MI-MUC-1 netOGlyc-4.0.0.13 CARBOHYD 492 492 0.954859 . .#POSITIVE

MI-MUC-1 netOGlyc-4.0.0.13 CARBOHYD 498 498 0.829118 . .#POSITIVE

MI-MUC-1 netOGlyc-4.0.0.13 CARBOHYD 501 501 0.750755 . .#POSITIVE

MI-MUC-1 netOGlyc-4.0.0.13 CARBOHYD 502 502 0.921907 . .#POSITIVE

MI-MUC-1 netOGlyc-4.0.0.13 CARBOHYD 526 526 0.735765 . .#POSITIVE

MI-MUC-1 netOGlyc-4.0.0.13 CARBOHYD 528 528 0.702523 . .#POSITIVE

MI-MUC-1 netOGlyc-4.0.0.13 CARBOHYD 531 531 0.828727 . .#POSITIVE

MI-MUC-1 netOGlyc-4.0.0.13 CARBOHYD 543 543 0.817629 . .#POSITIVE

MI-MUC-1 netOGlyc-4.0.0.13 CARBOHYD 544 544 0.507423 . .#POSITIVE

MI-MUC-1 netOGlyc-4.0.0.13 CARBOHYD 549 549 0.583719 . .#POSITIVE

MI-MUC-1 netOGlyc-4.0.0.13 CARBOHYD 554 554 0.57686 . . #POSITIVE

MI-MUC-1 netOGlyc-4.0.0.13 CARBOHYD 556 556 0.28177 . .

MI-MUC-1 netOGlyc-4.0.0.13 CARBOHYD 577 577 0.912351 . .#POSITIVE

MI-MUC-1 netOGlyc-4.0.0.13 CARBOHYD 582 582 0.939305 . .#POSITIVE

MI-MUC-1 netOGlyc-4.0.0.13 CARBOHYD 587 587 0.905679 . .#POSITIVE

MI-MUC-1 netOGlyc-4.0.0.13 CARBOHYD 592 592 0.854154 . .#POSITIVE

MI-MUC-1 netOGlyc-4.0.0.13 CARBOHYD 595 595 0.921684 . .#POSITIVE

MI-MUC-1 netOGlyc-4.0.0.13 CARBOHYD 596 596 0.967708 . .#POSITIVE

MI-MUC-1 netOGlyc-4.0.0.13 CARBOHYD 597 597 0.959701 . .#POSITIVE

MI-MUC-1 netOGlyc-4.0.0.13 CARBOHYD 604 604 0.972394 . .#POSITIVE

MI-MUC-1 netOGlyc-4.0.0.13 CARBOHYD 605 605 0.954821 . .#POSITIVE

MI-MUC-1 netOGlyc-4.0.0.13 CARBOHYD 612 612 0.969856 . .#POSITIVE

MI-MUC-1 netOGlyc-4.0.0.13 CARBOHYD 614 614 0.966698 . .#POSITIVE

MI-MUC-1 netOGlyc-4.0.0.13 CARBOHYD 617 617 0.965174 . .#POSITIVE

MI-MUC-1 netOGlyc-4.0.0.13 CARBOHYD 618 618 0.984631 . .#POSITIVE

MI-MUC-1 netOGlyc-4.0.0.13 CARBOHYD 619 619 0.947119 . .#POSITIVE

MI-MUC-1 netOGlyc-4.0.0.13 CARBOHYD 621 621 0.966085 . .#POSITIVE

MI-MUC-1 netOGlyc-4.0.0.13 CARBOHYD 631 631 0.896996 . .#POSITIVE

MI-MUC-1 netOGlyc-4.0.0.13 CARBOHYD 633 633 0.883264 . .#POSITIVE

MI-MUC-1 netOGlyc-4.0.0.13 CARBOHYD 634 634 0.878875 . .#POSITIVE

MI-MUC-1 netOGlyc-4.0.0.13 CARBOHYD 643 643 0.913193 . .#POSITIVE

MI-MUC-1 netOGlyc-4.0.0.13 CARBOHYD 645 645 0.871768 . .#POSITIVE

MI-MUC-1 netOGlyc-4.0.0.13 CARBOHYD 650 650 0.885781 . .#POSITIVE

MI-MUC-1 netOGlyc-4.0.0.13 CARBOHYD 651 651 0.898979 . .#POSITIVE

MI-MUC-1 netOGlyc-4.0.0.13 CARBOHYD 655 655 0.86922 . . #POSITIVE

MI-MUC-1 netOGlyc-4.0.0.13 CARBOHYD 673 673 0.662141 . .#POSITIVE

MI-MUC-1 netOGlyc-4.0.0.13 CARBOHYD 674 674 0.73327 . . #POSITIVE

MI-MUC-1 netOGlyc-4.0.0.13 CARBOHYD 678 678 0.749985 . .#POSITIVE

MI-MUC-1 netOGlyc-4.0.0.13 CARBOHYD 684 684 0.464281 . .

MI-MUC-1 netOGlyc-4.0.0.13 CARBOHYD 699 699 0.383299 . .

MI-MUC-1 netOGlyc-4.0.0.13 CARBOHYD 712 712 0.610225 . .#POSITIVE

MI-MUC-1 netOGlyc-4.0.0.13 CARBOHYD 713 713 0.439872 . .

MI-MUC-1 netOGlyc-4.0.0.13 CARBOHYD 720 720 0.333442 . .

MI-MUC-1 netOGlyc-4.0.0.13 CARBOHYD 726 726 0.411797 . .

MI-MUC-1 netOGlyc-4.0.0.13 CARBOHYD 734 734 0.377791 . .

MI-MUC-1 netOGlyc-4.0.0.13 CARBOHYD 735 735 0.403976 . .

MI-MUC-1 netOGlyc-4.0.0.13 CARBOHYD 741 741 0.38129 . .

MI-MUC-1 netOGlyc-4.0.0.13 CARBOHYD 750 750 0.327778 . .

MI-MUC-1 netOGlyc-4.0.0.13 CARBOHYD 755 755 0.245404 . .

MI-MUC-1 netOGlyc-4.0.0.13 CARBOHYD 775 775 0.788258 . .#POSITIVE

MI-MUC-1 netOGlyc-4.0.0.13 CARBOHYD 776 776 0.690706 . .#POSITIVE

MI-MUC-1 netOGlyc-4.0.0.13 CARBOHYD 789 789 0.512451 . .#POSITIVE

MI-MUC-1 netOGlyc-4.0.0.13 CARBOHYD 792 792 0.53778 . . #POSITIVE

MI-MUC-1 netOGlyc-4.0.0.13 CARBOHYD 797 797 0.308743 . .

MI-MUC-1 netOGlyc-4.0.0.13 CARBOHYD 801 801 0.321722 . .

MI-MUC-1 netOGlyc-4.0.0.13 CARBOHYD 812 812 0.732875 . .#POSITIVE

MI-MUC-1 netOGlyc-4.0.0.13 CARBOHYD 813 813 0.461234 . .

MI-MUC-1 netOGlyc-4.0.0.13 CARBOHYD 820 820 0.930707 . .#POSITIVE

MI-MUC-1 netOGlyc-4.0.0.13 CARBOHYD 821 821 0.956578 . .#POSITIVE

MI-MUC-1 netOGlyc-4.0.0.13 CARBOHYD 825 825 0.949086 . .#POSITIVE

MI-MUC-1 netOGlyc-4.0.0.13 CARBOHYD 826 826 0.906093 . .#POSITIVE

MI-MUC-1 netOGlyc-4.0.0.13 CARBOHYD 831 831 0.981869 . .#POSITIVE

MI-MUC-1 netOGlyc-4.0.0.13 CARBOHYD 834 834 0.971004 . .#POSITIVE

MI-MUC-1 netOGlyc-4.0.0.13 CARBOHYD 835 835 0.937525 . .#POSITIVE

MI-MUC-1 netOGlyc-4.0.0.13 CARBOHYD 837 837 0.656039 . .#POSITIVE

MI-MUC-1 netOGlyc-4.0.0.13 CARBOHYD 846 846 0.692614 . .#POSITIVE

MI-MUC-1 netOGlyc-4.0.0.13 CARBOHYD 850 850 0.96427 . . #POSITIVE

MI-MUC-1 netOGlyc-4.0.0.13 CARBOHYD 857 857 0.975808 . .#POSITIVE

MI-MUC-1 netOGlyc-4.0.0.13 CARBOHYD 858 858 0.976829 . .#POSITIVE

MI-MUC-1 netOGlyc-4.0.0.13 CARBOHYD 860 860 0.973297 . .#POSITIVE

MI-MUC-1 netOGlyc-4.0.0.13 CARBOHYD 862 862 0.975172 . .#POSITIVE

MI-MUC-1 netOGlyc-4.0.0.13 CARBOHYD 864 864 0.986138 . .#POSITIVE

MI-MUC-1 netOGlyc-4.0.0.13 CARBOHYD 867 867 0.985829 . .#POSITIVE

MI-MUC-1 netOGlyc-4.0.0.13 CARBOHYD 873 873 0.952064 . .#POSITIVE

MI-MUC-1 netOGlyc-4.0.0.13 CARBOHYD 877 877 0.974157 . .#POSITIVE

MI-MUC-1 netOGlyc-4.0.0.13 CARBOHYD 878 878 0.938261 . .#POSITIVE

MI-MUC-1 netOGlyc-4.0.0.13 CARBOHYD 885 885 0.941862 . .#POSITIVE

MI-MUC-1 netOGlyc-4.0.0.13 CARBOHYD 888 888 0.982471 . .#POSITIVE

MI-MUC-1 netOGlyc-4.0.0.13 CARBOHYD 893 893 0.97093 . . #POSITIVE

MI-MUC-1 netOGlyc-4.0.0.13 CARBOHYD 894 894 0.960839 . .#POSITIVE

MI-MUC-1 netOGlyc-4.0.0.13 CARBOHYD 896 896 0.974378 . .#POSITIVE

MI-MUC-1 netOGlyc-4.0.0.13 CARBOHYD 897 897 0.975139 . .#POSITIVE

MI-MUC-1 netOGlyc-4.0.0.13 CARBOHYD 903 903 0.968 . . #POSITIVE

MI-MUC-1 netOGlyc-4.0.0.13 CARBOHYD 909 909 0.760034 . .#POSITIVE

MI-MUC-1 netOGlyc-4.0.0.13 CARBOHYD 915 915 0.880702 . .#POSITIVE

MI-MUC-1 netOGlyc-4.0.0.13 CARBOHYD 919 919 0.850232 . .#POSITIVE

MI-MUC-1 netOGlyc-4.0.0.13 CARBOHYD 923 923 0.853564 . .#POSITIVE

MI-MUC-1 netOGlyc-4.0.0.13 CARBOHYD 925 925 0.787259 . .#POSITIVE

MI-MUC-1 netOGlyc-4.0.0.13 CARBOHYD 926 926 0.769834 . .#POSITIVE

MI-MUC-1 netOGlyc-4.0.0.13 CARBOHYD 931 931 0.782971 . .#POSITIVE

MI-MUC-1 netOGlyc-4.0.0.13 CARBOHYD 939 939 0.835015 . .#POSITIVE

MI-MUC-1 netOGlyc-4.0.0.13 CARBOHYD 945 945 0.892449 . .#POSITIVE

MI-MUC-1 netOGlyc-4.0.0.13 CARBOHYD 947 947 0.822574 . .#POSITIVE

MI-MUC-1 netOGlyc-4.0.0.13 CARBOHYD 948 948 0.911897 . .#POSITIVE

MI-MUC-1 netOGlyc-4.0.0.13 CARBOHYD 949 949 0.949207 . .#POSITIVE

MI-MUC-1 netOGlyc-4.0.0.13 CARBOHYD 956 956 0.904625 . .#POSITIVE

MI-MUC-1 netOGlyc-4.0.0.13 CARBOHYD 957 957 0.968945 . .#POSITIVE

MI-MUC-1 netOGlyc-4.0.0.13 CARBOHYD 958 958 0.822153 . .#POSITIVE

MI-MUC-1 netOGlyc-4.0.0.13 CARBOHYD 961 961 0.802555 . .#POSITIVE

MI-MUC-1 netOGlyc-4.0.0.13 CARBOHYD 966 966 0.903032 . .#POSITIVE

MI-MUC-1 netOGlyc-4.0.0.13 CARBOHYD 969 969 0.878482 . .#POSITIVE

MI-MUC-1 netOGlyc-4.0.0.13 CARBOHYD 972 972 0.788562 . .#POSITIVE

MI-MUC-1 netOGlyc-4.0.0.13 CARBOHYD 975 975 0.891574 . .#POSITIVE

MI-MUC-1 netOGlyc-4.0.0.13 CARBOHYD 977 977 0.794427 . .#POSITIVE

MI-MUC-1 netOGlyc-4.0.0.13 CARBOHYD 983 983 0.712115 . .#POSITIVE

MI-MUC-1 netOGlyc-4.0.0.13 CARBOHYD 987 987 0.90911 . . #POSITIVE

MI-MUC-1 netOGlyc-4.0.0.13 CARBOHYD 992 992 0.932934 . .#POSITIVE

MI-MUC-1 netOGlyc-4.0.0.13 CARBOHYD 993 993 0.93147 . . #POSITIVE

MI-MUC-1 netOGlyc-4.0.0.13 CARBOHYD 997 997 0.818766 . .#POSITIVE

MI-MUC-1 netOGlyc-4.0.0.13 CARBOHYD 999 999 0.81275 . . #POSITIVE

MI-MUC-1 netOGlyc-4.0.0.13 CARBOHYD 1005 1005 0.659338 . .#POSITIVE

MI-MUC-1 netOGlyc-4.0.0.13 CARBOHYD 1008 1008 0.769499 . .#POSITIVE

MI-MUC-1 netOGlyc-4.0.0.13 CARBOHYD 1009 1009 0.930106 . .#POSITIVE

MI-MUC-1 netOGlyc-4.0.0.13 CARBOHYD 1015 1015 0.948845 . .#POSITIVE

MI-MUC-1 netOGlyc-4.0.0.13 CARBOHYD 1016 1016 0.946118 . .#POSITIVE

MI-MUC-1 netOGlyc-4.0.0.13 CARBOHYD 1018 1018 0.967266 . .#POSITIVE

MI-MUC-1 netOGlyc-4.0.0.13 CARBOHYD 1020 1020 0.968469 . .#POSITIVE

MI-MUC-1 netOGlyc-4.0.0.13 CARBOHYD 1022 1022 0.978283 . .#POSITIVE

MI-MUC-1 netOGlyc-4.0.0.13 CARBOHYD 1023 1023 0.992572 . .#POSITIVE

MI-MUC-1 netOGlyc-4.0.0.13 CARBOHYD 1024 1024 0.959876 . .#POSITIVE

MI-MUC-1 netOGlyc-4.0.0.13 CARBOHYD 1026 1026 0.933473 . .#POSITIVE

MI-MUC-1 netOGlyc-4.0.0.13 CARBOHYD 1029 1029 0.9749 . . #POSITIVE

MI-MUC-1 netOGlyc-4.0.0.13 CARBOHYD 1030 1030 0.98014 . . #POSITIVE

MI-MUC-1 netOGlyc-4.0.0.13 CARBOHYD 1032 1032 0.973297 . .#POSITIVE

MI-MUC-1 netOGlyc-4.0.0.13 CARBOHYD 1035 1035 0.939267 . .#POSITIVE

MI-MUC-1 netOGlyc-4.0.0.13 CARBOHYD 1037 1037 0.954967 . .#POSITIVE

MI-MUC-1 netOGlyc-4.0.0.13 CARBOHYD 1038 1038 0.988965 . .#POSITIVE

MI-MUC-1 netOGlyc-4.0.0.13 CARBOHYD 1047 1047 0.95347 . . #POSITIVE

MI-MUC-1 netOGlyc-4.0.0.13 CARBOHYD 1048 1048 0.940379 . .#POSITIVE

MI-MUC-1 netOGlyc-4.0.0.13 CARBOHYD 1051 1051 0.922902 . .#POSITIVE

MI-MUC-1 netOGlyc-4.0.0.13 CARBOHYD 1053 1053 0.901725 . .#POSITIVE

MI-MUC-1 netOGlyc-4.0.0.13 CARBOHYD 1058 1058 0.820634 . .#POSITIVE

MI-MUC-1 netOGlyc-4.0.0.13 CARBOHYD 1059 1059 0.720019 . .#POSITIVE

MI-MUC-1 netOGlyc-4.0.0.13 CARBOHYD 1068 1068 0.672368 . .#POSITIVE

MI-MUC-1 netOGlyc-4.0.0.13 CARBOHYD 1073 1073 0.918043 . .#POSITIVE

MI-MUC-1 netOGlyc-4.0.0.13 CARBOHYD 1074 1074 0.891174 . .#POSITIVE

MI-MUC-1 netOGlyc-4.0.0.13 CARBOHYD 1075 1075 0.945963 . .#POSITIVE

MI-MUC-1 netOGlyc-4.0.0.13 CARBOHYD 1076 1076 0.937492 . .#POSITIVE

MI-MUC-1 netOGlyc-4.0.0.13 CARBOHYD 1081 1081 0.953917 . .#POSITIVE

MI-MUC-1 netOGlyc-4.0.0.13 CARBOHYD 1083 1083 0.992148 . .#POSITIVE

MI-MUC-1 netOGlyc-4.0.0.13 CARBOHYD 1084 1084 0.987908 . .#POSITIVE

MI-MUC-1 netOGlyc-4.0.0.13 CARBOHYD 1086 1086 0.962288 . .#POSITIVE

MI-MUC-1 netOGlyc-4.0.0.13 CARBOHYD 1087 1087 0.990738 . .#POSITIVE

MI-MUC-1 netOGlyc-4.0.0.13 CARBOHYD 1091 1091 0.99074 . . #POSITIVE

MI-MUC-1 netOGlyc-4.0.0.13 CARBOHYD 1092 1092 0.962858 . .#POSITIVE

MI-MUC-1 netOGlyc-4.0.0.13 CARBOHYD 1093 1093 0.960465 . .#POSITIVE

MI-MUC-1 netOGlyc-4.0.0.13 CARBOHYD 1094 1094 0.98515 . . #POSITIVE

MI-MUC-1 netOGlyc-4.0.0.13 CARBOHYD 1095 1095 0.957215 . .#POSITIVE

MI-MUC-1 netOGlyc-4.0.0.13 CARBOHYD 1098 1098 0.978191 . .#POSITIVE

MI-MUC-1 netOGlyc-4.0.0.13 CARBOHYD 1104 1104 0.979561 . .#POSITIVE

MI-MUC-1 netOGlyc-4.0.0.13 CARBOHYD 1105 1105 0.911563 . .#POSITIVE

MI-MUC-1 netOGlyc-4.0.0.13 CARBOHYD 1107 1107 0.904836 . .#POSITIVE

MI-MUC-1 netOGlyc-4.0.0.13 CARBOHYD 1108 1108 0.969384 . .#POSITIVE

MI-MUC-1 netOGlyc-4.0.0.13 CARBOHYD 1109 1109 0.922784 . .#POSITIVE

MI-MUC-1 netOGlyc-4.0.0.13 CARBOHYD 1123 1123 0.32826 . .
