# Supplementary material for: Knockdown of a mucin‐like gene in Meloidogyne incognita (Nematoda) decreases attachment of endospores of Pasteuria penetrans to the infective juveniles and reduces nematode fecundity
Source: Mol Plant Pathol. 2018 Oct 22;19(11):2370–83. doi: 10.1111/mpp.12704 (PMC6638177; doi:10.1111/mpp.12704)
Supplement: Supplementary file 6 — Table S2 List of mucin‐like proteins of Caenorhabditis elegans. [file MPP-19-2370-s006.docx]

**Table S2:** List of mucin-like proteins of *C. elegans*

| **Sl** | **WormBase Accession No.** | **Protein sequence length (bp)** | **% (serine + threonine)** |
| --- | --- | --- | --- |
| 1 | ZC178.2 | 1459 | 51.26 |
| 2 | K06A9.1b | 2232 | 50.04 |
| 3 | H02F09.3 | 1275 | 49.80 |
| 4 | F59A6.3 | 786 | 49.74 |
| 5 | H43E16.1 | 1203 | 39.81 |
| 6 | T19D12.1 | 1844 | 35.52 |
| 7 | FIG-1b | 2892 | 31.43 |
| 8 | C12D12.1a | 769 | 30.68 |
| 9 | DCT-17a | 739 | 28.41 |
| 10 | DPY6 | 1254 | 35.32 |
| 11 | CLEC185 | 504 | 34.52 |
| 12 | Y43F8C.16 | 370 | 40.27 |
| 13 | C26G2.2 | 1148 | 23.08 |
| 14 | T01D3.1b | 3226 | 19.18 |
| 15 | T21D12.11 | 709 | 36.95 |
